# DeNeRD: high-throughput detection of neurons for brain-wide analysis with deep learning

**DOI:** 10.1038/s41598-019-50137-9

**Published:** 2019-09-25

**Authors:** Asim Iqbal, Asfandyar Sheikh, Theofanis Karayannis

**Affiliations:** 1 Laboratory of Neural Circuit Assembly, Brain Research Institute (HiFo), UZH, Zürich, Switzerland; 2 Neuroscience Center Zurich (ZNZ), UZH/ETH Zurich, Zürich, Switzerland

**Keywords:** Development of the nervous system, Learning algorithms

## Abstract

Mapping the structure of the mammalian brain at cellular resolution is a challenging task and one that requires capturing key anatomical features at the appropriate level of analysis. Although neuroscientific methods have managed to provide significant insights at the micro and macro level, in order to obtain a whole-brain analysis at a cellular resolution requires a meso-scopic approach. A number of methods can be currently used to detect and count cells, with, nevertheless, significant limitations when analyzing data of high complexity. To overcome some of these constraints, we introduce a fully automated Artificial Intelligence (AI)-based method for whole-brain image processing to **De**tect **Ne**urons in different brain **R**egions during **D**evelopment (*DeNeRD*). We demonstrate a high performance of our deep neural network in detecting neurons labeled with different genetic markers in a range of imaging planes and imaging modalities.

## Introduction

Similar to other disciplines, neuroscience has entered the realm of ‘big data’, which brings forth the need for the development of methods that can enable researchers to explore their datasets in an unbiased and high-throughput manner. To allow for the application of comprehensive approaches in the study of the structure and function of the nervous system, a series of technological advancements are required, both at the hardware and software end. There has been a significant advancement of hardware development in the last couple of decades to record the activity of thousands of brain cells at a time through multichannel recording probes^1^ or large field of view fluorescent microscopes^2^, although the cells are usually confined to a given brain region. At the same time, big consortia and prime institutes world-wide e.g. Allen Brain Institute^3^, Blue Brain^4^, and Human Brain Project^5^ are producing high-throughput genetic and structural imaging data that capture and visualize many different types of neurons in the whole brain with spatially precise single-cell-resolution. Although many efforts have been made for providing a number for cell count in the entire brain^6^, these methods rely on different stereology techniques^7^, each of which has its strengths and weaknesses. Importantly, most approaches analyze a small area of brain and then extrapolate to the entire volume, assuming a normal Poisson distribution of cells, or use a non-histological method to count all the cells, but lose crucial information on the 3D position of the cells, as well as their type^8,9^. Recent advances in computational methods to identify individual cells in defined regions have provided a powerful tool to perform high-throughput analysis of cell numbers *in situ*^10,11^, but these methods are limited in scope due to a large variation of size, shape, and density of neurons in different regions of the brain.

To overcome such significant drawbacks there is a need to develop and implement unbiased high-throughput approaches that allow for any labeled cell in a given brain region or even the whole brain to be counted, while preserving the exact cell location. Deep Learning (DL) methods are gaining increasing attention in the biological and health sciences. These approaches are used in order to provide fast and comprehensive screening for a change in biological samples over time or in healthy versus potentially diseased human data^12,13^, but so far their use in mouse brain samples is sparse^14–16^. The advantage of AI-based methods over traditional computer vision-based techniques in object detection tasks is their power to capture the variance in an object’s structures without updating the set of hyperparameters for every given data sample. Such an approach is key to analyzing complete brains, owing to the existence of large diversity in scale, morphology and intensity of neuronal elements within and across different brain samples. Although, efforts have been made to detect cells using machine learning-based methods in the past, their performance has been limited to a specific tissue sample^17–20^.

In this study we develop a deep neural network-based method to detect neurons expressing a variety of genetic markers and captured with different imaging modalities during the course of mammalian brain development.

## Results

### A novel method for neuron detection using a Deep Neural Network

**Figure 1:**
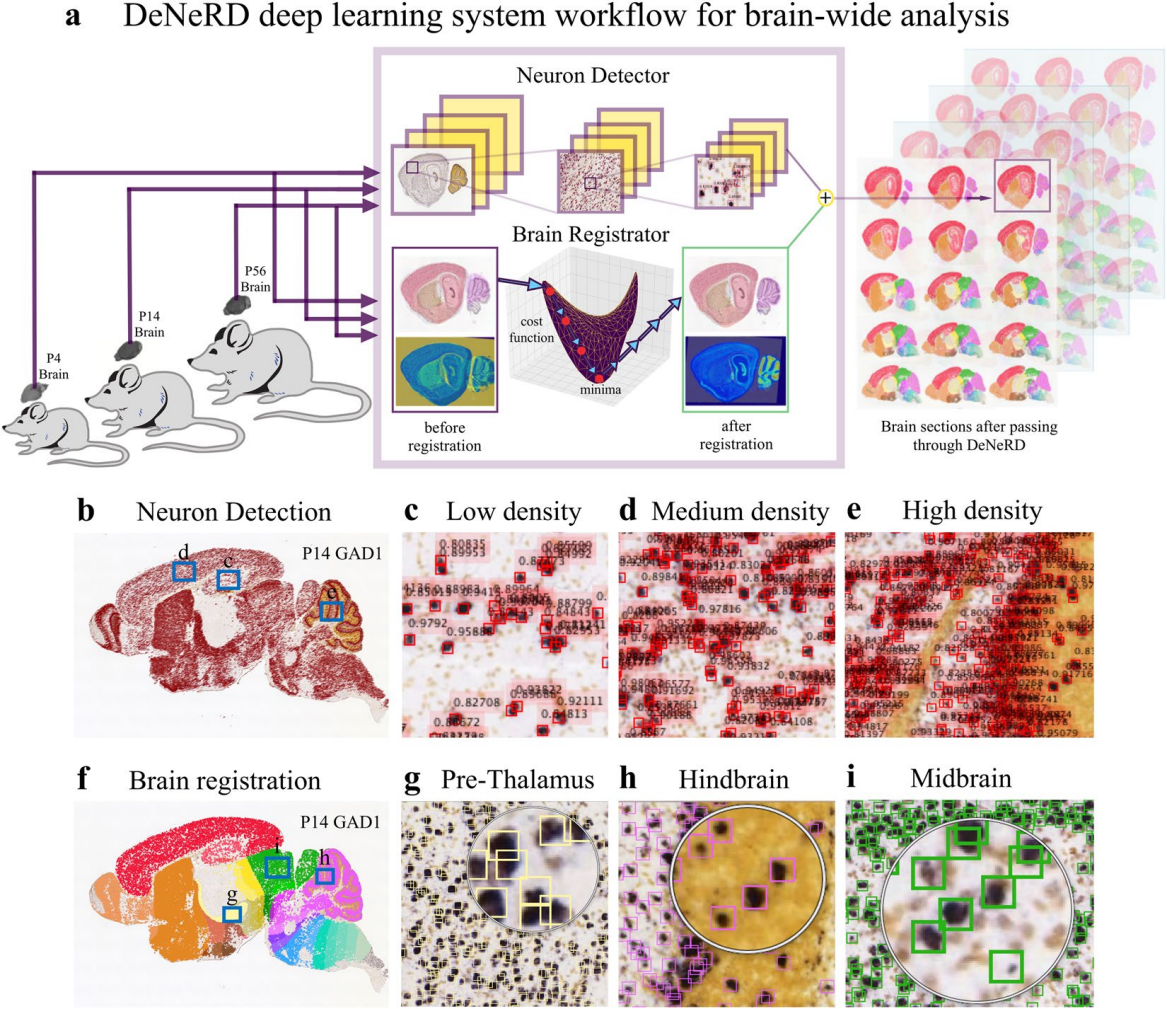
Workflow of the deep learning-based system, ***DeNeRD***. (**a**) Block diagram showing the input of brain images from various postnatal (P) time points that are processed by the *Neuron Detector* (top) unit. Each brain image is serially passed through the Faster Regions with Convolutional Neural Network (R-CNN)-based architecture, which detects and labels the neurons present in the entire brain section image. The same image is also passed through the *Brain Registrator* (bottom) unit, which automatically registers it with the Allen developing mouse brain atlas. Automated registration is performed through optimization of an affine transformation algorithm. The outputs of the *Neuron Detector* and the *Brain Registrator* are then combined to label all the detected neurons in the brain regions color-coded in the Allen mouse brain atlas. The output on the right side shows the clusters of neurons in different brain regions in both lateral and medial brain section images. (**b**–**e**) The output of the *Neuron Detector* on a P14 GAD1 sample mouse brain image is shown. The blue boxes delineate the zoomed-in images in c-e, where the detection of labeled neurons with their classification scores over the bounding boxes can be seen for different neuronal density areas. (**f**–**i**) The same P14 GAD1 brain section is passed through the *Brain Registrator* module, where the bounding boxes of detected neurons are colored with respect to the Allen brain atlas regions. (**g**–**i**) The performance of the *Neuron Detector* is shown at three randomly selected zoomed-in brain regions (pre-thalamus, hindbrain and midbrain). The original brain sections are obtained from^3^. Image credit: Allen Institute.

**Figure 2:**
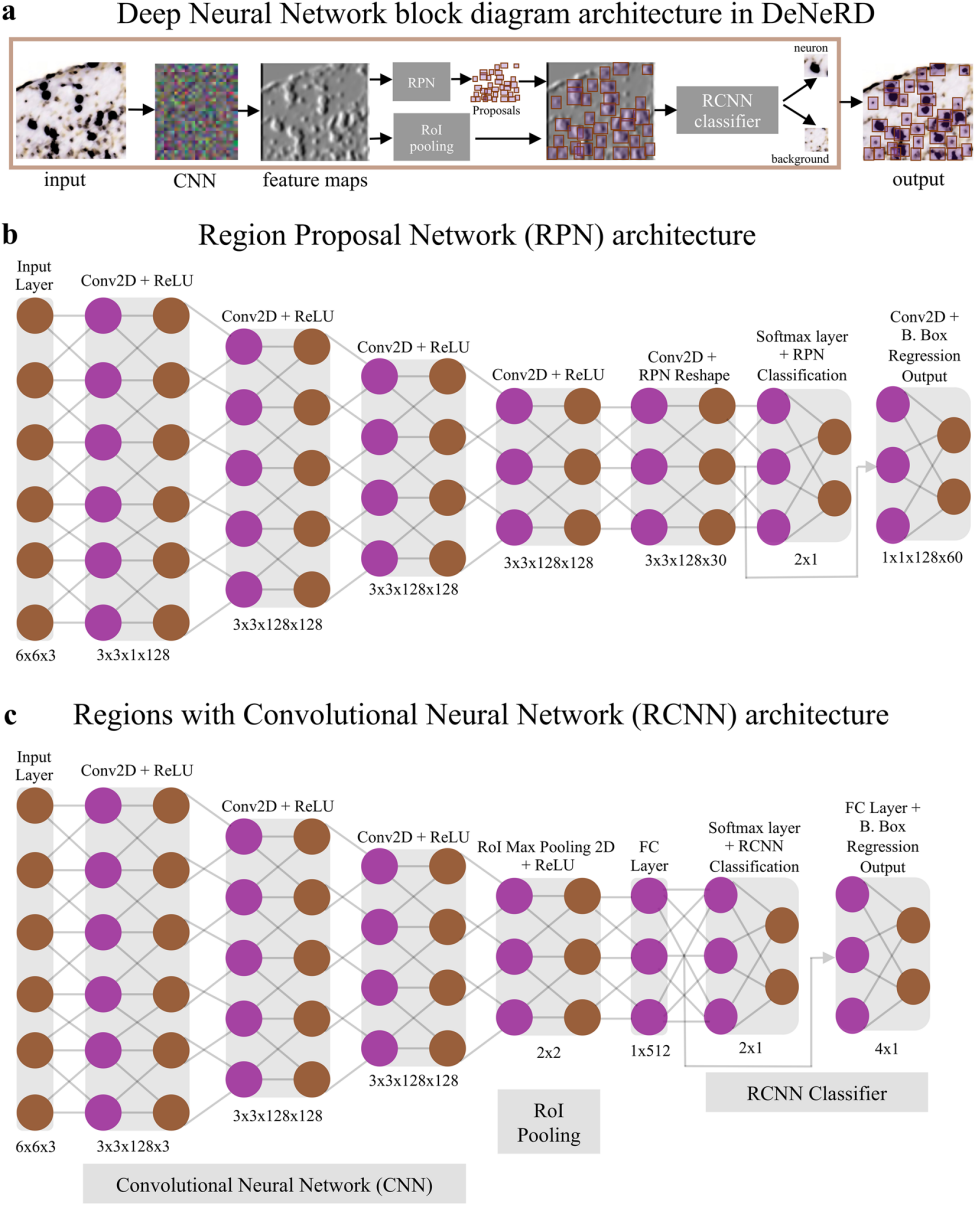
The Deep Neural Network architecture in the *DeNeRD* (*Neuron Detector*) pipeline for neuron detection. (**a**) The block diagram of Faster R-CNN in *DeNeRD*. A small brain section image is used as input that goes through a series of convolutional stages, RPN (Region Proposal Network), ROI (Region of Interest) Pooling and RCNN classifier. The outputs of RPN and ROI Pooling are combined and then fed as input to the RCNN classifier, which distinguishes the neurons from the background class. (**b**) RPN Architecture with convolutional, ReLU (Rectified Linear Unit) and pooling layers. (**c**) RCNN architecture with convolutional, ReLU, pooling and fully connected layers. The purple and brown colored nodes represent the different layers in the deep neural network which have functionally distinct roles in the feature processing of the input brain section images.

To expedite brain-wide analysis of neuronal distributions in brain sections *in situ*, we introduce here a Deep Learning method (*DeNeRD* - Detection of Neurons for Brain-wide analysis with Deep Learning), which is based on the state-of-the-art in object detection network called Faster Regions with Convolutional Neural Network (Faster RCNN)^21^ (Fig. 1a). *DeNeRD* takes images of mouse brain sections as input and after some pre-processing, it detects the neuronal populations in the pre-registered brain areas as shown in Fig. 1b–i. The architecture of our deep neural network is demonstrated in Fig. 2 with a step-by-step processing of an input brain section through convolutional stages. The detailed structure of the architecture is explained in the Methods section.

In order to test our network, we used three common brain markers: CaMKIIa, GAD1 and VGAT. These brain sections were taken from the online publicly available datasets at Allen Institute’s Online Public Resource (OPR)^3^. These markers label the largest and the second largest population of neurons in the mammalian brain. CaMKIIa labels excitatory cells, whereas GAD1 is the enzyme that regulates the production of GABA and VGAT, the transporter responsible for packing it into vesicles (hence both label inhibitory cells).

Since the deep neural network needs to go through a training session, we first had to generate the training (ground-truth) data. To expedite this process, we constructed a Simple Graphical User Interface (*SiGUI*) software, as shown in Supplementary Fig. S1, through which human experts annotated the bounding boxes on top of the neurons (Supplementary Fig. S2). Users can freely scroll towards left and right in the directory to jump on different brain images and draw and remove bounding boxes by selecting the “draw/remove” bounding box options from the right panel of the GUI. The intensity-thresholded (expression) versions of the *in situ* brain images were used as a reference to differentiate the real neural signal from the background noise (Supplementary Fig. S3). It is interesting to observe that the deep neural network is able to learn the features of neuronal structure during the procedure of training (Supplementary Fig. S4).

**Figure 3:**
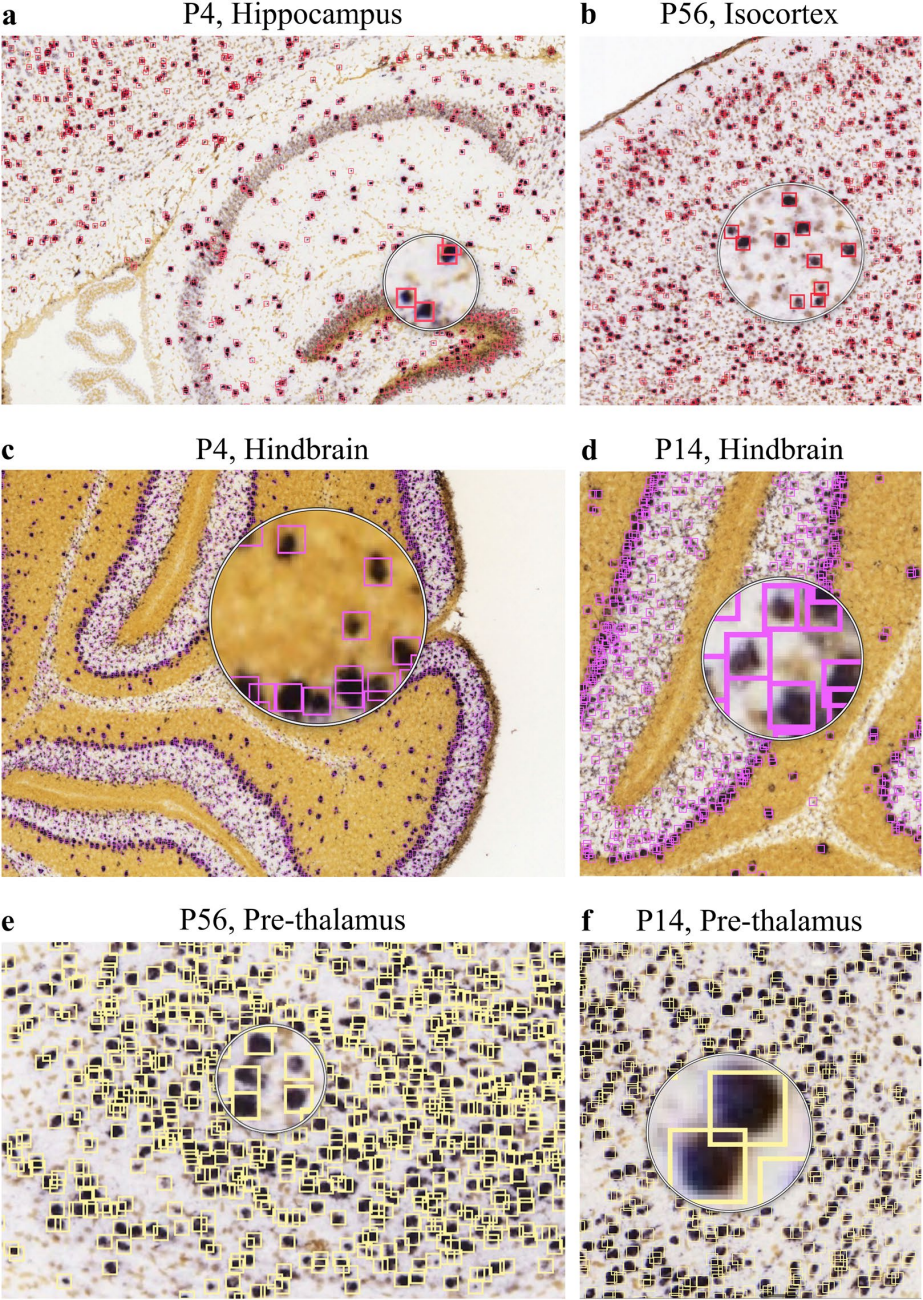
*DeNeRD* is shown detecting, counting and localizing neurons (different color bounding boxes depending on the region) of different shapes, sizes and intensities with a variety of backgrounds. Neuron detection in various brain regions with different neural densities at three postnatal (P) ages is shown on example images of *in*-*situ* hybridized (ISH) brain sections for GAD1. (**a**,**b**) The trained deep neural network detects neurons in cortical regions (P4-hippocampus and P56-isocortex). (**c**,**d**) Performance of deep neural network in detecting neurons in the hindbrain region of P4 and P14 mouse brain sections is shown. (**e**,**f**) Deep neural network is able to localize the neurons in pre-thalamus region of P56 and P14 mouse brains. The original brain sections are obtained from^3^. Image credit: Allen Institute.

Using CaMKIIa, GAD1 and VGAT and focusing on different brain regions across three different post-natal (P) developmental time points (P4, P14 and P56), we explored the power of our method to capture the large variation in expression pattern, neuronal shape and size. Figure 3 demonstrates the performance of *DeNeRD* in detecting neurons in the neural dataset of GAD1 and VGAT *in situ* hybridized (ISH) brain sections. These markers cover about 20% of all cortical neurons in the adult mouse cortex, hence presenting themselves as a reasonably challenging detection task for the deep neural network. We generated a dataset of 210 human-annotated images, where 1/2 of them (randomly selected) are used for training and the other 1/2 for testing purpose (see also Methods). We show that *DeNeRD* performs well in detecting inhibitory cells, with a mean average-precision (mAP) score of 0.9.

In order to further explore the performance of *DeNeRD*, we generated a ground-truth dataset of human annotated neurons in 140 images labeled with fluorescently-tagged CaMKIIa (converted into grayscale; see Methods), a marker that accounts for about 80% of cortical neurons. These brain sections are also obtained from the Allen Institute’s OPR. Detecting neurons in this dataset is a bigger challenge for *DeNeRD* due to a very low signal-to-noise ratio since the neuronal population is highly dense in most of the brain regions, which can lead to more cells overlapping that also have a large variation in size with lower background contrast. We, therefore, trained the deep neural network on 2/3 of randomly selected images from this dataset and tested its performance on the remaining 1/3. We observed a mean average-precision score of 0.75, with the mean precision of 0.87. However, to avoid any bias, no intensity and detection thresholding is applied here prior to feeding the testing data to the DNN. This may result in detection of low intensity neural signal, which can also be considered as background noise in some cases.

**Figure 4:**
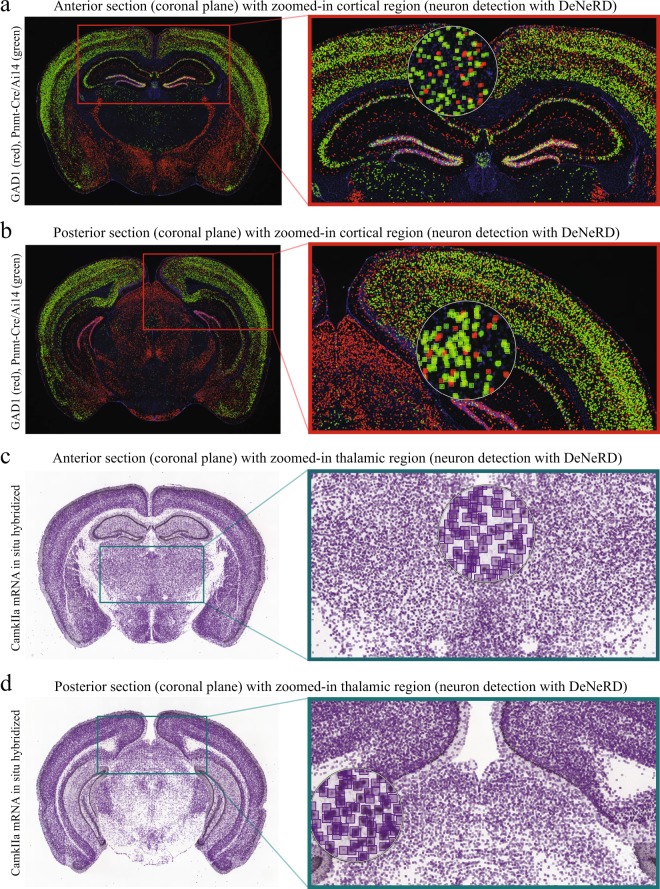
Performance of *DeNeRD* in detecting neurons labeled by a variety of brain markers and obtained via different imaging modalities at a coronal plane. (**a**) The performance of *DeNeRD* is shown in detecting neurons in a coronal section from the anterior part of a postnatal (P) 57 mouse’s brain which are fluorescently labeled for GAD1 tdTomato (red) and Pnmt-Cre/Ai14 (green). The detections of the deep neural network can be visualized as red (tdTomato) and green (Pnmt-Cre/Ai14) bounding boxes on top of neurons. The red box is the area shown as zoomed-in on the right side. (**b**) The performance of the method in detecting neurons in a posterior section from the mouse brain fluorescently labeled for GAD1 tdTomato (red) and Pnmt-Cre/Ai14 (green) is shown. Zoomed-in image from the CA1/subiculum hippocampal region is shown with neuron detection on the right side. (**c**) An anterior section from an adult brain labeled for CamkIIa by mRNA *in situ* hybridization is shown with neuron detection. Performance of the deep neural network in detecting excitatory neurons in the thalamus (purple bounding boxes) is shown on the right side. (**d**) A posterior section from the CamkIIa brain is shown with neuron detection. Performance of the deep neural network-based method in detecting neurons in the brainstem is showcased on the right side (zoomed). The original brain sections are obtained from^3^. Image credit: Allen Institute.

**Figure 5:**
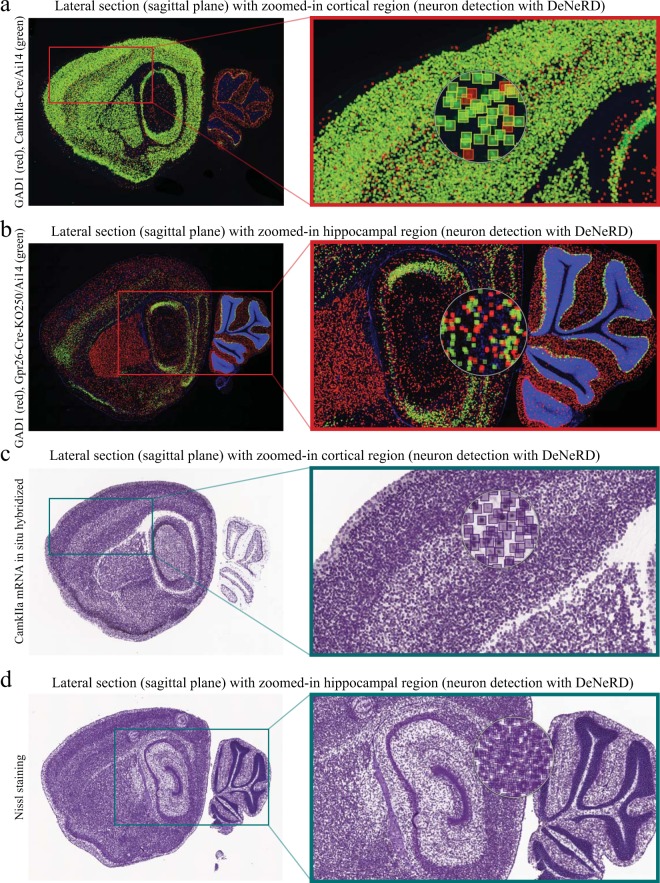
Assessing the performance of *DeNeRD* in detecting neurons in images of adult brain sections labeled neurons with a variety of brain markers taken with bright field and fluorescent microscopy at a sagittal plane. (**a**) The results obtained with *DeNeRD* in detecting a variety of neurons fluorescently labeled with GAD1 tdTomato (red) and CamkIIa-Cre/Ai14 (green) in a lateral image of a sagittal postnatal (P) 77 female mouse brain section. The red box is the area shown as zoomed-in on the right side. Further zoom is shown with the detected cells and their bounding boxes in the granular and infragranular cortical layers. (**b**) Performance of the Deep Neural Network (DNN) in detecting neurons labeled for GAD1 tdTomato (red) and Gpr26-Cre-KO250/Ai14 (green) in a P28 female brain section is shown. Zoom in on hippocampus and cerebellum is shown with the performance of deep neural network in neuron detection. (**c**) Lateral section from a CamkIIa-stained adult brain, labelling pyramidal neurons with the DNN detections on top. Zoomed-in sample is shown with purple bounding boxes for neuron detection in cortical region. (**d**) Nissl-stained adult (P56) brain section, labeling all neurons is shown, together with the DNN detections (purple bounding boxes) on top. Zoomed-in image is shown with the performance of deep neural network in detecting neurons in hippocampal region. The original brain sections are obtained from^3^. Image credit: Allen Institute.

To further challenge the performance of our method we used other Allen Institute images, from sections processed also for fluorescence *in situ* hybridization (FISH): GAD1-tdTomato(red)-Pnmt-Cre-Ai14(green), GAD1-tdTomato(red)-CamkIIa-Cre-Ai14(green), GAD1-tdTomato(red)-Gpr26-Cre-KO250-Ai14(green), as well as Nissl. These images contain fluorescently-labeled cells in different colors (green and red), where each of them represents a different genetic marker (Figs 4 and 5), which, interestingly, the network has not been trained on. The network performs quite well in detecting neurons in these samples. The bounding boxes are colored as red, green and purple to visualize the detections in different neurons. In some cases (e.g. Figs 4c,d, 5c), DNN seems to detect a few extra neurons which can easily be avoided after intensity and detection thresholding. In terms of density, the biggest challenge for *DeNeRD* was detecting cells in Nissl-stained brain sections, because in principle it should label all neurons in the brain. Nevertheless, *DeNeRD* is still able to detect most, if not all, the labelled cells with a good coverage as shown in Fig. 5d. More importantly, although *DeNeRD* was trained on a single fluorescently labeled genetic marker, our results show that its performance is high even when applied on brain images that it has not been trained on, especially evident in the case of Nissl-stained brain sections.

### Comparison with alternative cell detection techniques

Currently, a number of approaches for large-scale cell quantitation exist with most of them using feature detection, intensity thresholding and region accumulation^22^. Although these methods perform reasonably well with sparse cell distributions, they are challenged when cells have a high density, as is the case of Nissl-stained brain sections or specific brain regions in our dataset (e.g. the reticular thalamic nucleus for GAD1). Moreover, different neuronal populations in the brain can vary in size by one or higher orders of magnitude. This, in fact, is also showcased when quantifying the cell body size of different types of GAD1-positive inhibitory cells in the cortex. One sub-population of inhibitory neurons labeled for Parvalbumin (PV) for example typically have large and irregular cell bodies, compared to another one labeled for Vasoactive Intestinal Peptide (VIP), which have significantly smaller and round-shaped somata. This can also be the case for the same kind of cells located in different regions, for example PV-positive Purkinje cells are quite morphologically distinct to PV-positive bistratified cells in the hippocampus.

**Figure 6:**
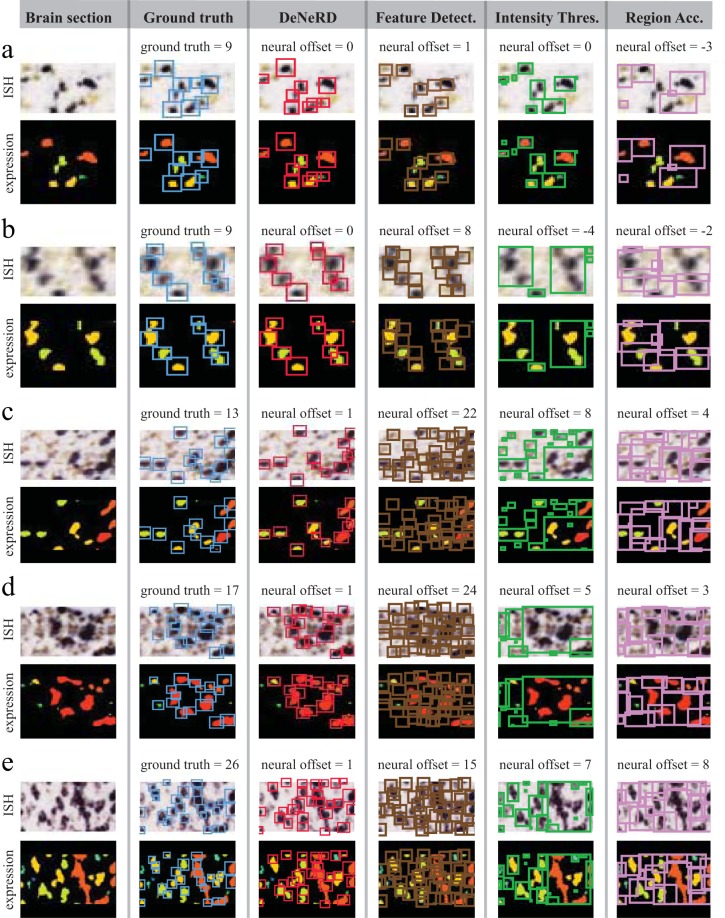
Performance comparison of *DeNeRD* with other commonly used neuron detection techniques. *DeNeRD* outperforms these methods in almost all the examples. For cases with <10 number of neurons in a given image (**a**,**b**), the other three methods show relatively low neural offset (*ground*_*truth*–*prediction*) score, but in examples containing >10 number of neurons (**c**–**e**), their neural offset score is increasingly high. Column 1 shows five cropped example images from brain sections with increasing neural density and complexity from top to bottom (mRNA *in*-*situ* hybridized with their expression images below each one). Column 2 shows the ground-truth (human annotated) bounding boxes (blue) on top of the neurons in brain images in column 1 with the number of neurons. Performance of *DeNeRD* in neuron detection on the given images in column 1 is shown in column 3 with red bounding boxes on top of the neuron detection along with the neural offset at the top. Performance of feature detection-based method is shown with neuron detection (brown bounding boxes) and neural offsets in column 4. Intensity thresholding-based method’s predictions are shown for neuron detection (green bounding boxes) along with the neural offsets in column 5. Region accumulation-based (watershed) algorithm is shown with the performance for neuron detection in pink bounding boxes with neural offsets in column 6.

**Figure 7:**
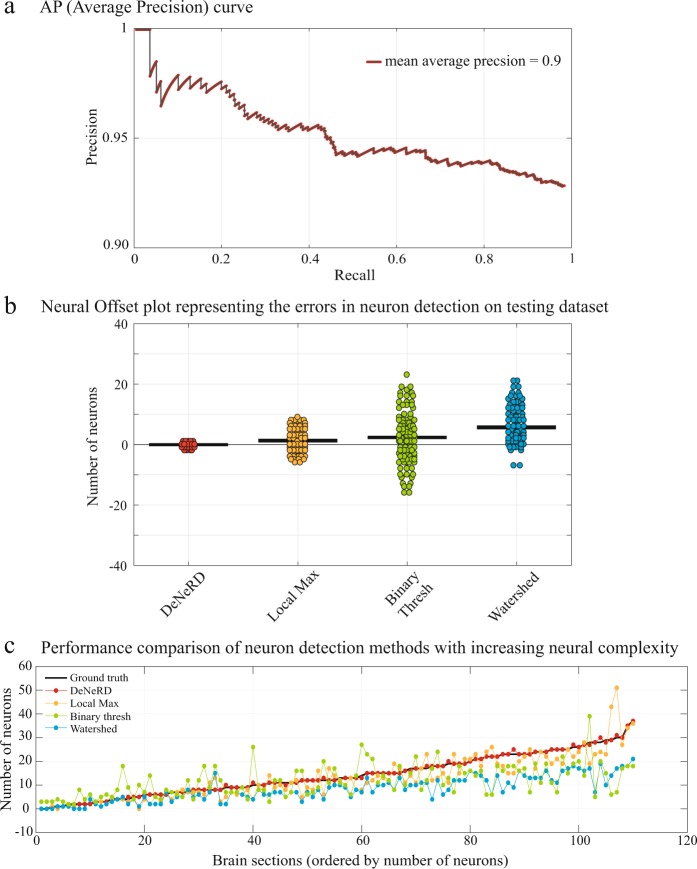
Quantitative performance of *DeNeRD* in neuron detection in comparison with other methods. (**a**) Precision-Recall curve of our method on the testing dataset. The deep neural network achieves a high score in detecting neurons with a mean average-precision (mAP) of 0.9. (**b**) Performance comparison of *DeNeRD* with commonly used neuron counting methods. *DeNeRD* gives the minimum offset (error between the ground-truth and prediction) on the testing dataset. The horizontal bar indicates the mean error on the complete testing dataset and each data point represents the error for a single brain section (n = 110). (**c**) *DeNeRD* performs well with an increase in complexity of neural structures and population density, whereas other methods drop off their performance. Brain sections are ranked according to their neural densities in the x-axis whereas the y-axis labels the number of neurons in the corresponding brain section.

In order to assess the power of our deep neural network-based method, we compared *DeNeRD*’s performance with some of the most commonly used cell detection methods. The result showed that our network outperforms other methods^22^ (Figs 6 and 7b,c) and provides a minimum neural offset with a high mean average-precision score (0.9) as demonstrated in Fig. 7a. The advantage of our deep learning-based neuron detection method over the existing methods is its power to learn the neuronal features from the labelled training data hence providing a generalized performance on testing datasets. In the compared methods, there exists a limitation in detecting neurons when they encounter a high degree of variability in the dataset. Feature detection method detects any object in the image with features similar to the neuronal shape without taking the neuronal signal intensity into account. In contrast, intensity thresholding and region accumulation methods are able to capture the neuronal signal but they are poor in detecting the neuronal features. All in all, the results show that our method is scalable to detect neurons in brain images that it has never “seen” before and is invariant to size, density and expression intensity of neurons in many different brain areas.

### Registering sections of the developing brain to a reference atlas

**Figure 8:**
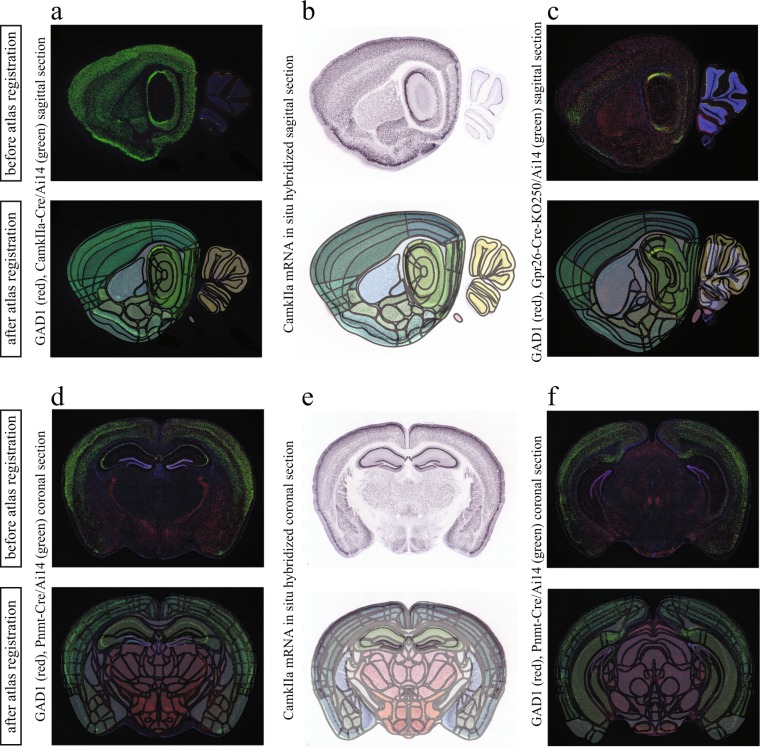
Automated registration of sagittal and coronal mouse brain (bright field and fluorescent) images to the detailed Allen brain atlas. Image registration is performed through the optimization of an affine transformation algorithm by adding a recurrent feedback loop that goes through multiple iterations until the minimum image registration error is achieved. (**a**–**f**) Sagittal or coronal brain sections obtained from different genetically modified animals are shown before and after automated image registration. The top rows show the brain images before and the bottom rows after image registration with the Allen brain mouse atlas.

To take full advantage of the power of *DeNeRD*, once it has been applied to a neural dataset to detect the cell populations of interest in whole brain sections, the following necessary step for researchers would be to extract the numbers and density per given area, region or sub-region. To achieve this, there is a need to register the processed image onto a reference atlas. A common way to apply image registration in an automated manner makes use of either rigid or non-rigid image transformation methods^23^. The Allen Institute provides the brain atlases at some post-natal time points in their database that are pre-registered only to their respective standard Nissl sections. In order to automatically register the brain image (ISH and FISH) sections, we first needed to transform the pre-registered Nissl brains to the given brain images and then apply their transformation matrices to the respective reference atlas. In this way, a given brain section (e.g. GAD1/VGAT) is registered to its corresponding reference atlas in the Allen Institute’s common coordinate framework. For automated registration, we optimized an affine transformation algorithm (elastix)^24^ by adding a recurrent feedback loop that goes through multiple iterations until the minimum error is reached. Supplementary Figs S5 and S6 show the automated registration procedure of a medial and lateral brain section. This strategy is visually represented in Supplementary Fig. S7a and it allowed for an optimal registration of the brain images of all post-natal ages as shown in Fig. 8 and Supplementary Fig. S7b. Additionally, we extract different areas in a given brain section based on the developmental origin, covering the whole brain. Supplementary Figs S8–S11 show the reference Nissl brains and their corresponding colored atlases that we can select for the purpose of automated registration. Furthermore, one can also extract the signal from sub-areas of the cortex, as well as individual cortical layers (Fig. 8) whenever a detailed atlas is available, which is only provided for the adult brain (P56) at Allen Institute. Once the step of atlas registration is achieved, one can subsequently apply neuron detection identification of the underlying cellular signal in brain regions and measure the neural densities of different neuronal types based on the expression of a marker in any given brain region during the course of brain development.

## Discussion

In this study, we have developed a deep neural network-based neuron detection method that works with a high performance on a variety of imaging planes, modalities and genetic markers. We show the detection of neurons with high precision on the most commonly used genetic markers such as CaMKIIa, GAD1 and VGAT. We demonstrate the performance of our method in a variety of imaging modalities such as *in situ* hybridization and fluorescence *in situ* hybridization, even for markers that the deep neural network has not been trained on such as Nissl. Using the power of *DeNeRD* and the atlas registration steps, one can ask a number of interesting questions, which include differences in the cell death, or differential neurogenesis of individual cell types (e.g. pyramidal and GABAergic cells) in various brain regions during development, and explore core principles of brain organization and development which would otherwise remain masked in the complexity and scale of the data. Beyond the neuroscience community, we believe that our method can be a valuable tool for the biology community in general.

## Methods

### Mouse brain sections

Images of brain sections used in this study were collected by mining the on-line open source database of the Allen Institute for Brain Science. According to the Institute’s website, their experimental data was collected in accordance with the guidelines and regulations of Allen Institute for Brain Science. Furthermore their experimental protocol for collecting the *in situ* hybridization tissue data was approved by the Allen Institute for Brain Science under Standard Operating Procedures (SOPs). The developing mouse brains were chosen at three unique post-natal time points: P4, P14 and P56, for two different markers: GAD1 (Glutamate decarboxylase 1) and VGAT (GABA vesicular transporter). Along with the *in situ* hybridized (ISH) brain sections, intensity-thresholded sections of the same brains were also imported which are utilized for ground-truth dataset generation. These ISH brain sections are 20 *μ*m thick, sectioned at 200 *μ*m apart – covering an entire hemisphere from lateral to medial. A couple of lateral and medial examples from adult mouse brain are shown in Supplementary Figs S5b and S6b, respectively. In total, six brains were utilized – a pair of GAD1 and VGAT brains at each developmental time point for the generation of inhibitory neural dataset. Similarly, two CamkIIa brains (P28+) were collected from the Allen Institute to generate the excitatory neural dataset.

### Neural dataset generation for training the deep neural network

A large dataset of six different brains is collected from Allen Brain’s OPR, in total 220 (P4 GAD1: 16 + 16, P4 VGAT: 18 + 18, P14 GAD1: 17 + 17, P14 VGAT: 19 + 19, P56 GAD1: 20 + 20 and P56 VGAT: 20 + 20) high resolution images. A pair of two brains from each post-natal development time points (P4, P14, P56) is selected. For each brain, both ISH and intensity-thresholded images are utilized. ISH images are used for the classification of neurons and the corresponding expression/intensity-thresholded images are used for applying the color threshold filter in order to differentiate the neural signal from the background. Initially, selected high-resolution brain sections are segmented into smaller images by dividing a brain section into 100 × 100 smaller sections, whereas each smaller image contains ~20 neurons. These small images are transferred to a specific directory, which is imported into the *SiGUI*. In total, 220 randomly selected sections are used for human annotation, where half of them (110) are randomly used for training and the other half (110) for testing purpose. Supplementary Fig. S2 shows the brain sections, followed by their expression versions in Supplementary Fig. S3. In case of CamkIIa brain dataset, we generated the ground-truth data on 140 brain sections that are taken from P28+ ages of mice from Allen Brain’s OPR. We converted these images into grayscale before training the deep neural network in order to test the performance of the network on different imaging modalities.

### Ground-truth labelling

A Simple Graphical User Interface (*SiGUI*) software is developed to produce the ground-truth data. Ground truth labels were generated by three human experts who annotated the bounding boxes on top of the neurons as shown in Supplementary Figs S1,S2. Users can freely scroll left and right in the directory to jump on different images and draw/remove bounding boxes by selecting the “draw/remove” bounding box options from the right panel of the GUI. The expression versions of the same brain sections were used as a reference to differentiate the real neural signal from background noise (Supplementary Fig. S3).

### Deep neural network architecture

The architecture of our network is inspired by the Faster RCNN^21^, as shown in Fig. 2. Designing of the network architecture, training and testing is performed in MATLAB. A four-step training procedure is applied that includes: (i) Training of the Region Proposal Network (RPN), (ii) Using the RPN from (i) to train a Fast RCNN, (iii) Retraining RPN by sharing weights with Fast RCNN of (ii). Finally, (iv) Retraining Fast RCNN using updated RPN. Epoch size of 500 is used with initial learning rate of 1 × 10^−5^ and 1 × 10^−6^ in stages (i-ii) and (iii-iv) respectively. Furthermore, box pyramid scale value of 1.2 is set, with positive and negative overlapping ranges of [0.51] and [00.5]. Number of box pyramidal levels are set to 15, with the minimum object size of [2.2]. For all the convolutional stages, a padding size of [1, 1, 1, 1] is selected, with [1, 1] as the size of strides, whereas a grid size of [2, 2] is set in the RoI pooling layer. Training is performed using NVIDIA Quadro P4000 GPU. Our network is trained by minimizing the multi-task loss which corresponds to each labeled Region of Interest, RoI (i.e. neuronal body), through a stochastic gradient descent algorithm^25^. Supplementary Fig. S4 shows the network learning features of neurons (weight vector) after training. Similar to^21^, loss of our network is described as following:

$$L={L}_{cls}+{L}_{reg}$$

Here, *L*_*cls*_ is the classification loss of neuron, calculated as a log loss for neuron *vs*. non-neuron classes, and *L*_*reg*_ is the regression loss of bounding box, where *L*_*cls*_ and *L*_*reg*_ are defined by following:

$$L({p}_{i},{q}_{i})=\frac{1}{{n}_{cls}}\sum _{i}{L}_{cls}({p}_{i},{p}_{i}^{\ast })+\lambda \frac{1}{{n}_{reg}}\sum _{i}{L}_{reg}({q}_{i},{q}_{i}^{\ast })$$

*p*_*i*_ = proposed probability of the *i*^th^ RoI (or anchor)

$${p}_{i}^{\ast }=\{\begin{array}{c}1,{p}_{i}\ge 0.7\\ 0,{p}_{i}\le 0.3\end{array}$$

*q*_*i*_ = bounding box coordinates of predicted RoI/anchor

*q*_i_* = bounding box coordinates of a positive anchor

*n*_*cls*_ = normalization parameter of classification loss, *L*_*cls*_

*n*_*reg*_ = normalization parameter of regression loss, *L*_*reg*_

*λ* = weight parameter

### Neural detector pipeline

Each brain section is serially processed by the neural detector. After some pre-processing, an input brain section (*B*) of size *p* × *q* pixels is divided into smaller brain sections (*b*_*n*_) by dividing *B* into *s* × *t* equal sections, here, *s* = *t* = 100. Zero padding is performed on each section in *b*_*n*_, hence the dimensions of a single image, *b*_*i*_, are increased to (*s* + 1 × + 1). Now each *b*_*i*_ (*i* = 1  → *n*) is passed through the deep neural network (Fig. 2a) which detects the neurons in each small section and returns the corresponding bounding boxes against each neural location. Finally, a binary image (*α*) of the same dimension (*s* + 1 × *t* + 1) as *b*_*i*_ is generated, where *α* ϵ {0, 1}. The center of each bounding box location in a given *b*_*i*_ is set to 1 in *α* and 0 otherwise. After removing the zero padding, *α* is stored with the corresponding iteration # *i*, and a similar process is repeated until the *n*th brain section. Once all the smaller sections (*b*_1→*n*_) are passed through the detector and the corresponding binary sections (α_1→*n*_) are produced, these binary sections are concatenated as following to create a single binary image (*A*) with neural locations as 1 s and background pixels as 0 s. The dimension of *A* will be the same as the input brain section, *p* × *q*.

$${A}_{(p\times q)}={\alpha }_{1}|{\alpha }_{2}|{\alpha }_{3}|{\alpha }_{4}\ldots |{\alpha }_{n}$$

### Brain registrator pipeline

For the purpose of brain registration, standard Nissl sections of P4, P14 and P56 mouse brains are also collected from Allen Institute’s OPR. For instance, lateral and medial Nissl sections of an adult brain are shown in Supplementary Figs S5c and S6c, respectively. Allen developing mouse brain reference atlases are pre-registered with these Nissl sections as shown in Supplementary Figs S5e and S6e, respectively.

Affine algorithm in elastix toolbox^24^ is optimized for the purpose of registration of brain sections. The affine transformation takes a fixed as well as a moving image as input and returns a transformed parameter map which can be applied to the moving image. The un-registered brain section (*B*) along with the corresponding registered Nissl section (*N*) are passed to the pipeline as fixed and moving images. *B* and *N* are first down-sampled by a reasonable ratio (*r*) such as to reduce the computational cost of brain registrator pipeline, without compromising the image quality of brain sections. *N* is first converted to gray scale, then both *B* and *N* are passed through a Gaussian filter (*B* × *g*(*μ*, *σ*) and *N* × *g*(*μ*, *σ*)) with *σ* = 1, to normalize brain sections with respect to the background noise. The corresponding reference atlas (*R*) of Nissl section is also down sampled to the same ratio, *r*.

After preprocessing, *N* is registered and transformed to the dimensions of *B* and the final metric score (error of registration/cost function) is calculated. The same procedure is repeated for *n* times and the final metric score is plotted against every registration step as shown in Supplementary Fig. S7a to find the minimum of error, here *n* = 20. Intermediate steps of registration for the first ten iterations in medial and lateral sagittal sections are shown in Supplementary Figs S5a and S6a respectively. The corresponding image, *N*, at the minimum of cost function is used for the purpose of registration. The transform parameter map is applied to the corresponding reference atlas, *R*. Supplementary Figs S5f and S6f shows the reference atlas, *R*, overlaid on top of the original brain section, *B*, after registration whereas Supplementary Figs S5d and S5d show the overlaid reference atlas before registration.

### Neural density measurement

Registered reference atlases (*R*_*i*_) of brains are collected from brain registrator, and binary neural images (*A*_*i*_) are imported from neural detector pipeline. Each brain region (*R*_*i*_) has a unique RGB color code, brain regions (*R*_*N*_) are filtered by their respective RGB codes and temporarily stored in the form of binary images where active *R*_*i*_ pixels are 1 s, and 0 s otherwise:

$${R}_{i}(x)=\{\begin{array}{c}1,\,x=[{r}_{i},{g}_{i},{b}_{i}]\\ 0,\,x\ne [{r}_{i},{g}_{i},{b}_{i}]\end{array}$$

Afterwards, neural density (*D*_*i*_) of a particular brain region (*R*_*i*_) can be calculated by taking its dot product with the binary neural image of a given brain section (*A*_*i*_) and normalizing it by dividing with the area of the brain region, $|{R}_{i}|$.

$${D}_{i}=\frac{{R}_{i}\cdot {A}_{i}}{|{R}_{i}|}$$

The same process is repeated for the rest of the brain regions (*R*_*N*-*1*_) on a given *B*_*i*_. Once, the neural density is measured for a single brain section (*A*_*i*_) then next brain section in the pipeline can be analyzed and so on until *A*_*N*_ for a particular mouse age, where *N* denotes the total number of brain regions.

### Software availability

The code and datasets generated and/or analyzed during the current study are available from the corresponding author on a reasonable request. *DeNeRD* is available online on Github (https://github.com/itsasimiqbal/DeNeRD) and Bitbucket (https://bitbucket.org/theolab/) for the neuroscience community.

## Supplementary information

Supplementary Information
