# Supplementary material for: DeNeRD: high-throughput detection of neurons for brain-wide analysis with deep learning
Source: Sci Rep. 2019 Sep 25;9:13828. doi: 10.1038/s41598-019-50137-9 (PMC6761257; doi:10.1038/s41598-019-50137-9)
Supplement: Supplementary file 1 — Supplementary Information [file 41598_2019_50137_MOESM1_ESM.pdf]

Supplementary Information

DeNeRD: High-throughput Detection of Neurons for Brain-wide analysis with Deep Learning

Asim Iqbal[1,2], Asfandyar Sheikh[1,3], Theofanis Karayannis[1,2]

[1]Laboratory of Neural Circuit Assembly, Brain Research Institute (HiFo), UZH
[2]Neuroscience Center Zurich (ZNZ), UZH/ETH Zurich
[3]Department of Information Technology and Electrical Engineering (D-ITET), ETH Zurich

*Correspondence should be addressed to T.K. (karayannis@hifo.uzh.ch)

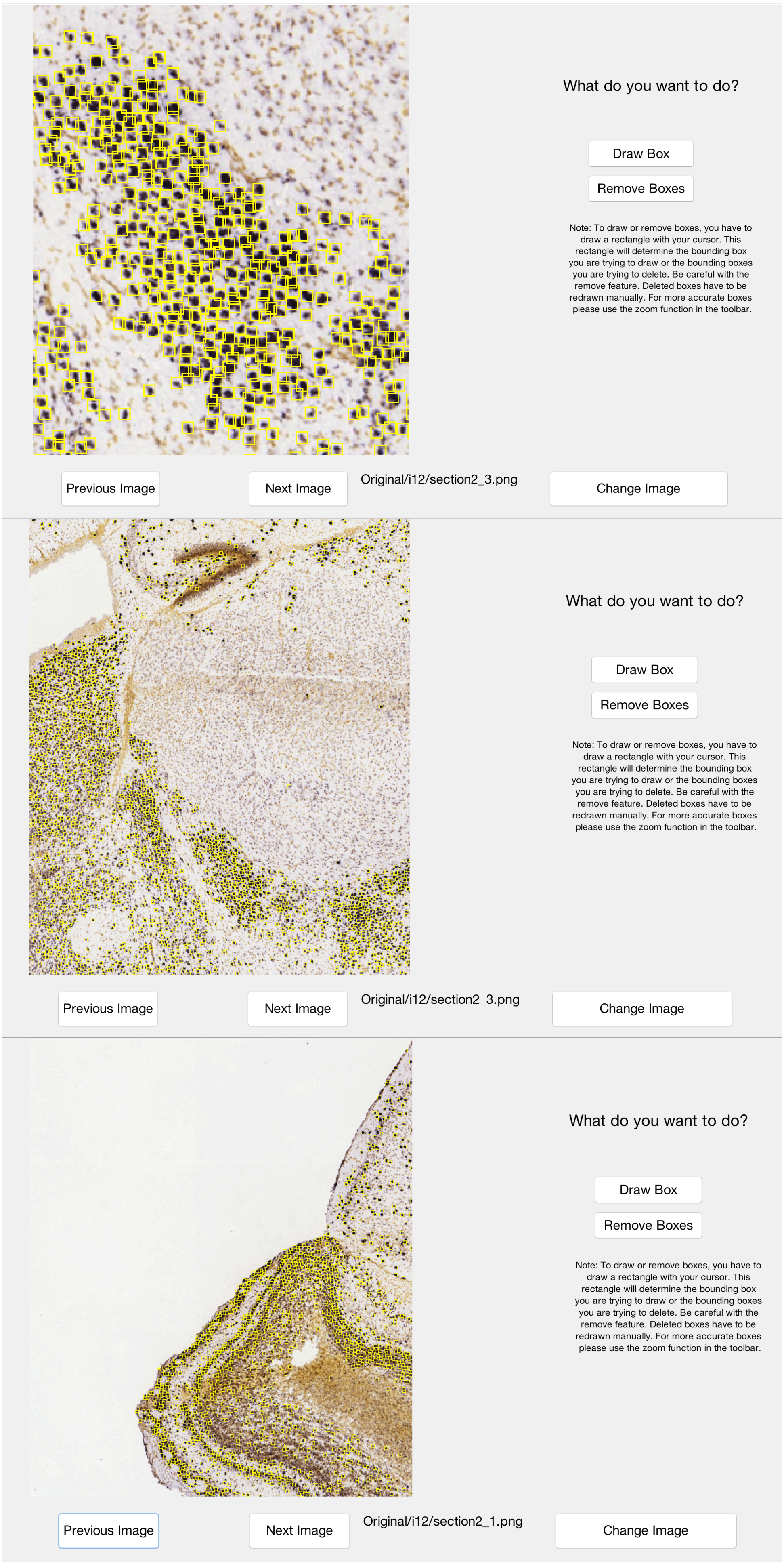

Supplementary Figure 1. Simple Graphical User Interface (SiGUI). Training data for the deep neural network to detect and localize neurons is generated by using SiGUI. Human user can simply draw/remove boxes on neurons, and go to the next/previous brain section with ease in the entire folder of brain datasets.

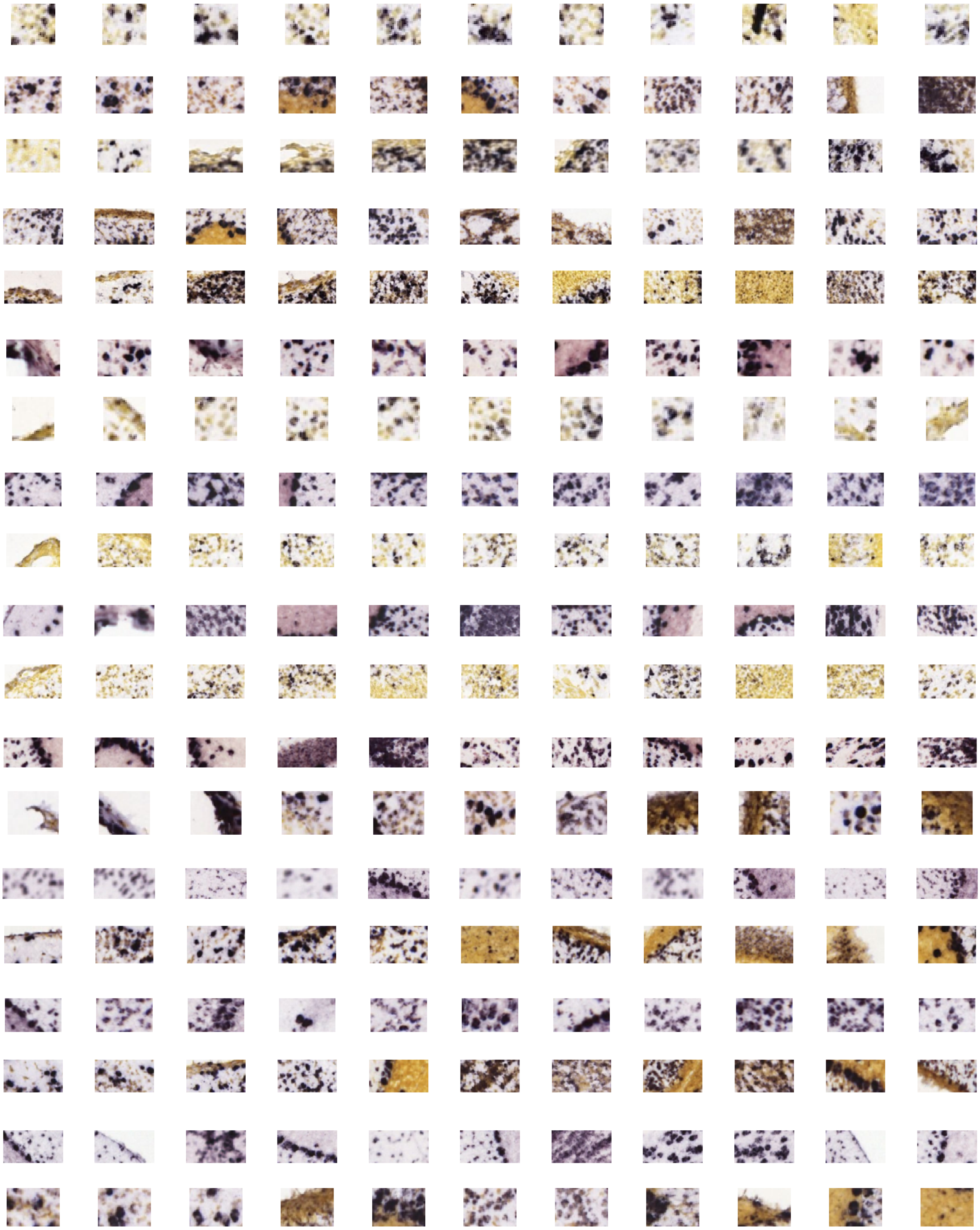

Supplementary Figure 2. ISH Brain sections to generate the ground-truth data for deep neural network. 220 brain sections are randomly chosen from six different brains at three developing ages (P4, P14 and P56) of GAD1 and VGAT markers. These brain sections contain samples from various brain regions e.g. cortex, hippocampus, hindbrain, midbrain, etc. in order to add diversity in the dataset.

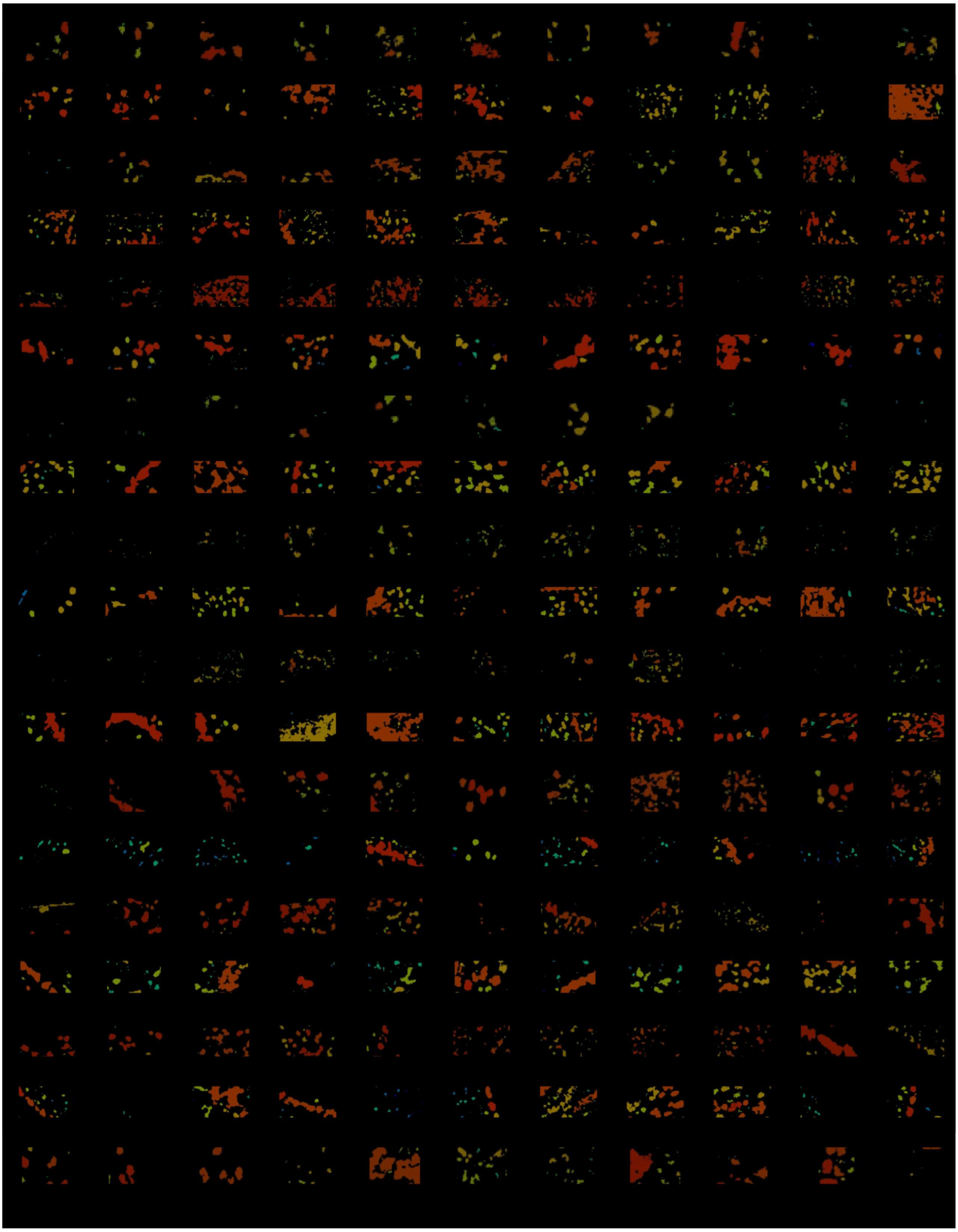

Supplementary Figure 3. Expression of brain sections to generate the ground-truth data for deep neural network. 220 samples (expression) of the same brain sections in Supplementary Fig. S2 are shown. The neural expression is shown as a heat map where red is the strongest signal and blue is the weakest. These expression images are utilized to filter the real neurons from the background noise. Furthermore, human annotation is performed by taking these expression samples as filters to differentiate between the real neural signal with background structures.

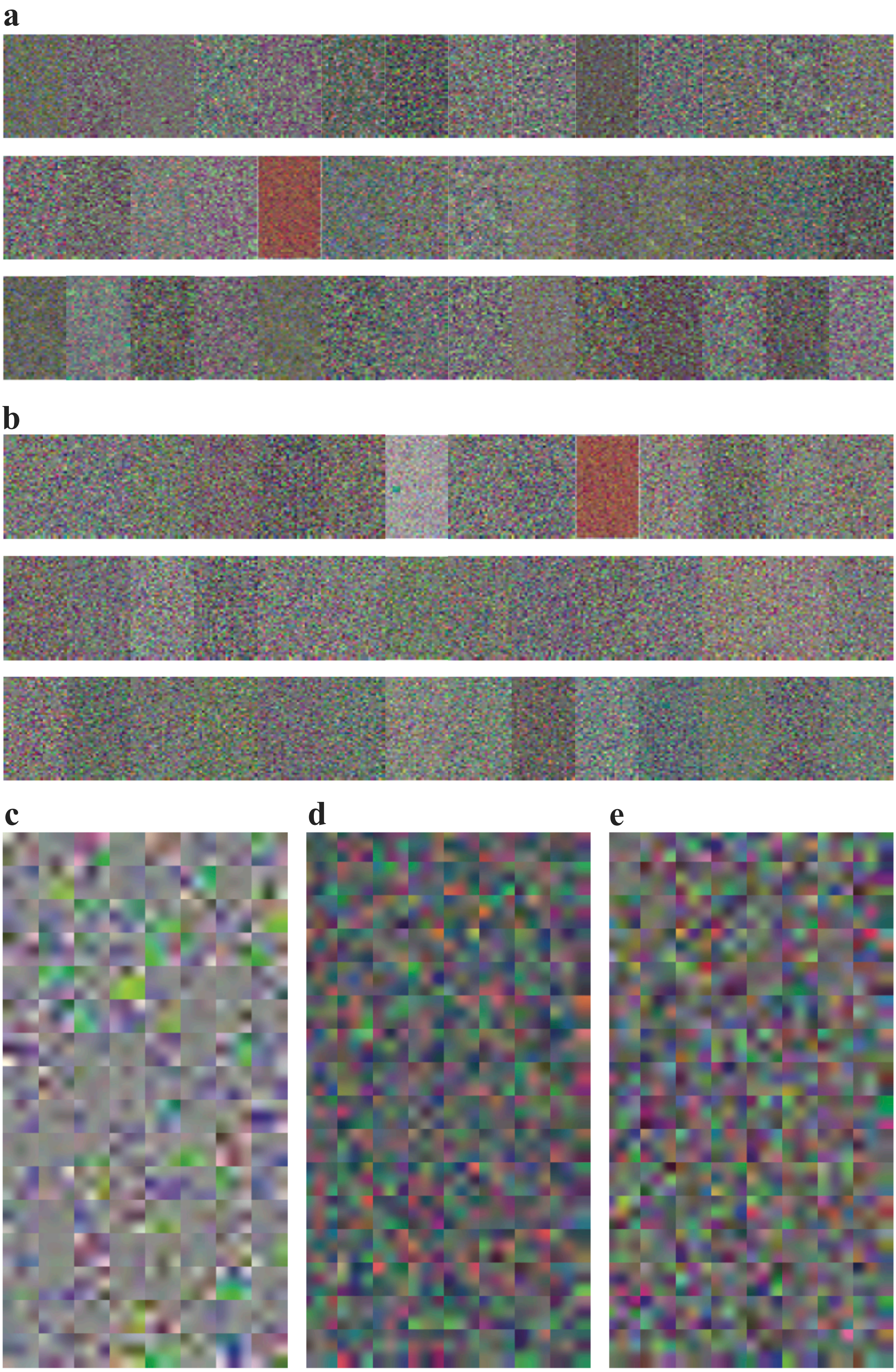

Supplementary Figure 4. Weight vectors of convolutional layers in DeNeRD.
a-c, Weight vectors of convolutional layers (conv4, conv6 and conv2) in Faster R-CNN-based architecture. c-d, A subset of weight vectors (highlighted in red in a-b) are shown. e, Weight vector of conv2 layer is visualised. It can be observed that network has learnt the features of neurons in the neural dataset after training (c-e) such as detecting boundaries of neurons as well as different structures and shapes.

a

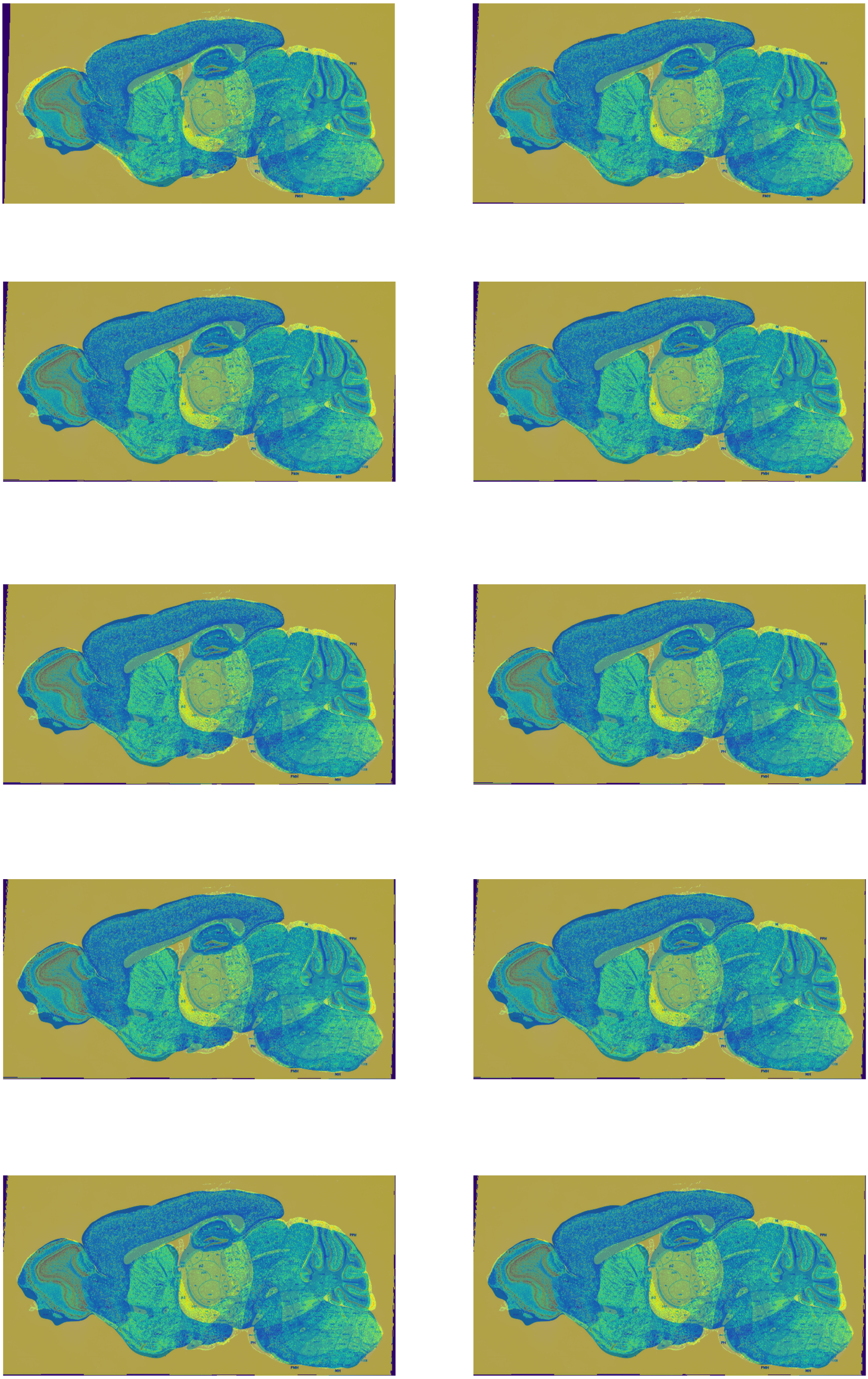

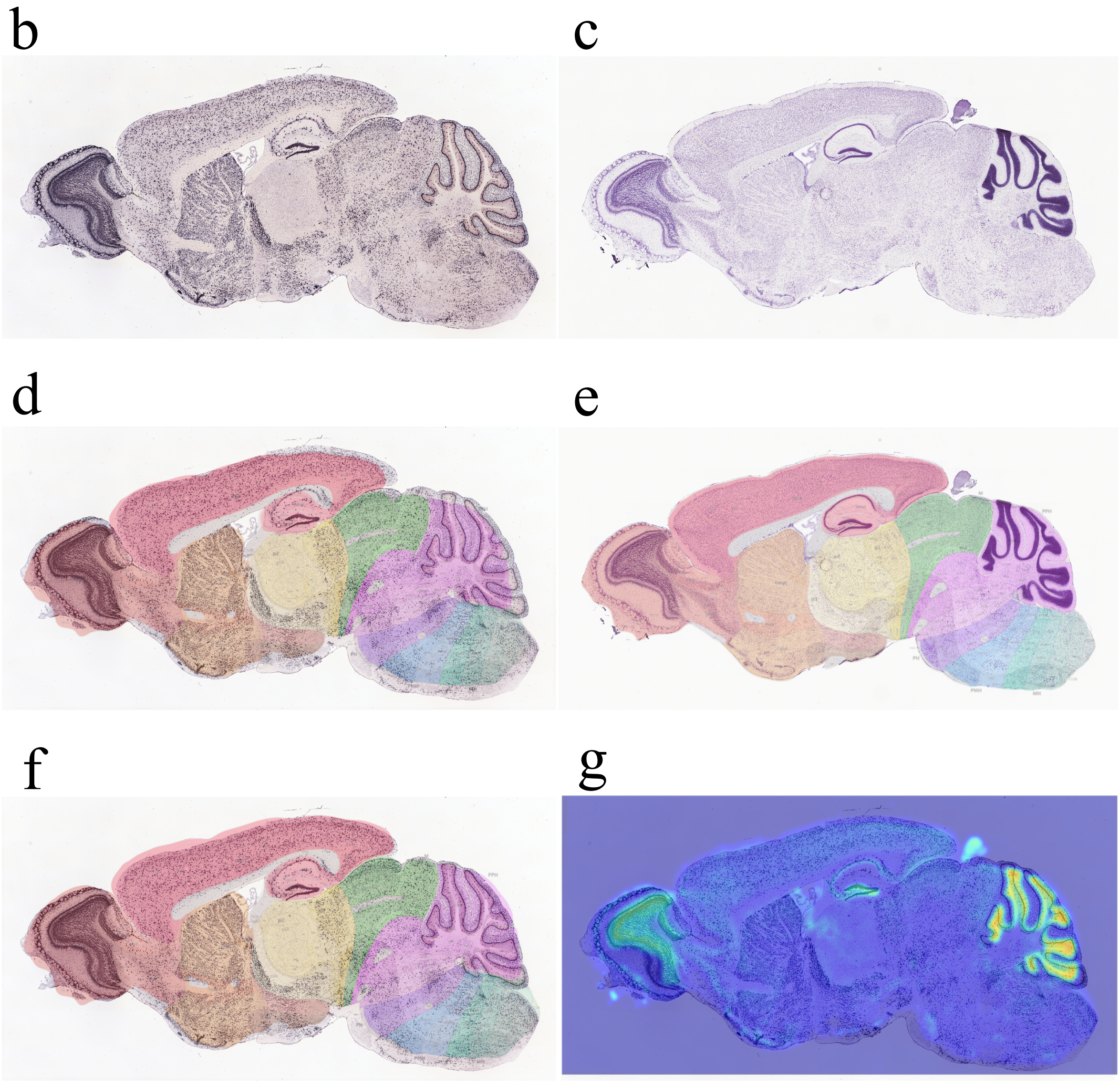

Supplementary Figure 5. Automatic registration of a medial brain section (sagittal) of an adult brain with Allen brain developing reference atlas. a) Figure shows the first ten iterations by optimization of affine transformation algorithm in ITK toolkit. **b)** Sample medial (sagittal) section of VGAT marker from P56 mouse brain. **c)** Nissl brain section against the corresponding brain section in (b) from the common coordinate framework in Allen brain atlas. **d)** Overlaid reference atlas on top of the brain section in (b) before registration. **e)** Overlaid reference atlas on top of the pre-registered Nissl brain section. **f)** Overlaid reference atlas on top of the VGAT brain section in (b) after registration. **g)** Overlaid VGAT brain section with transformed Nissl brain section after registration. The original brain sections are obtained from [3]. Image credit: Allen Institute.

a

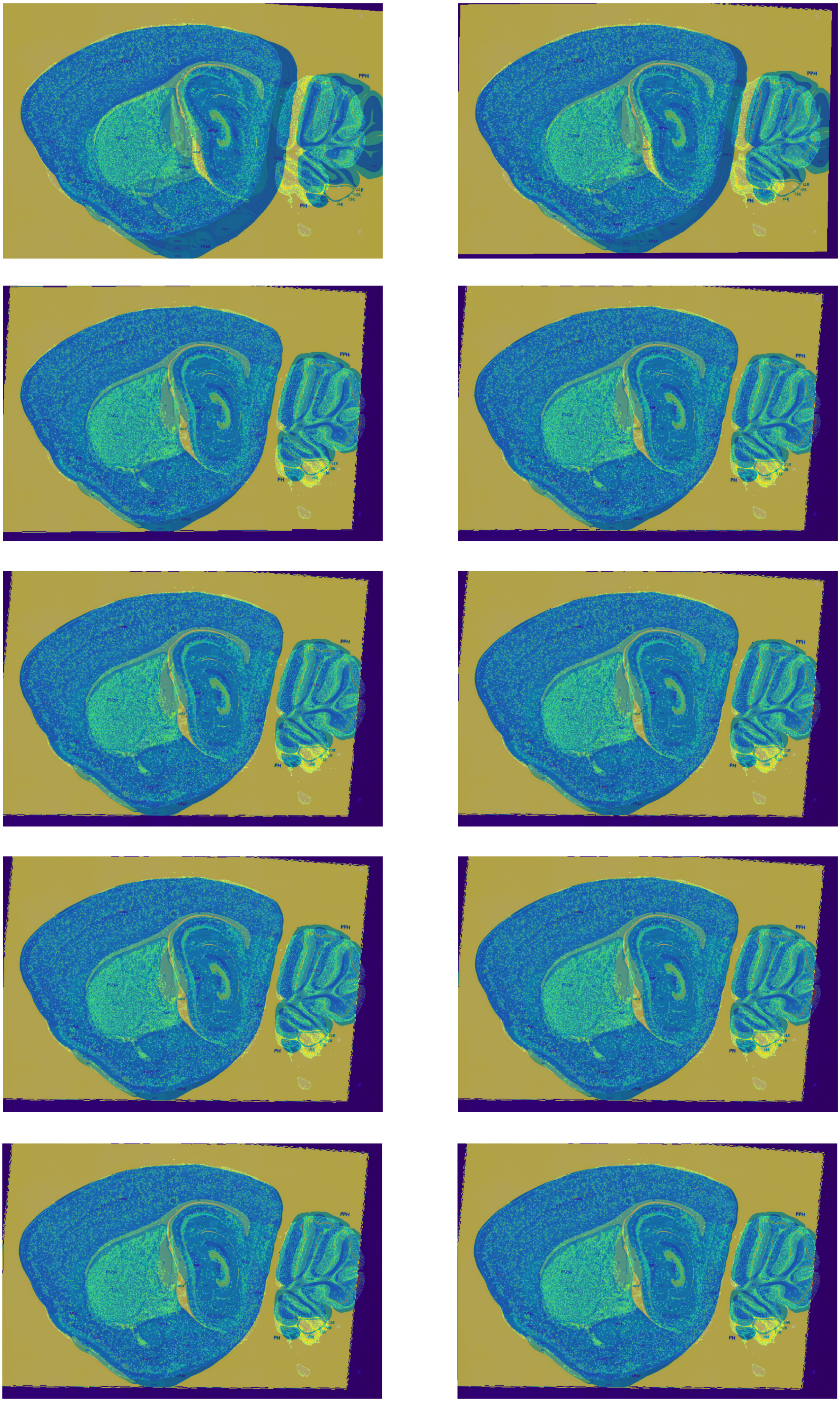

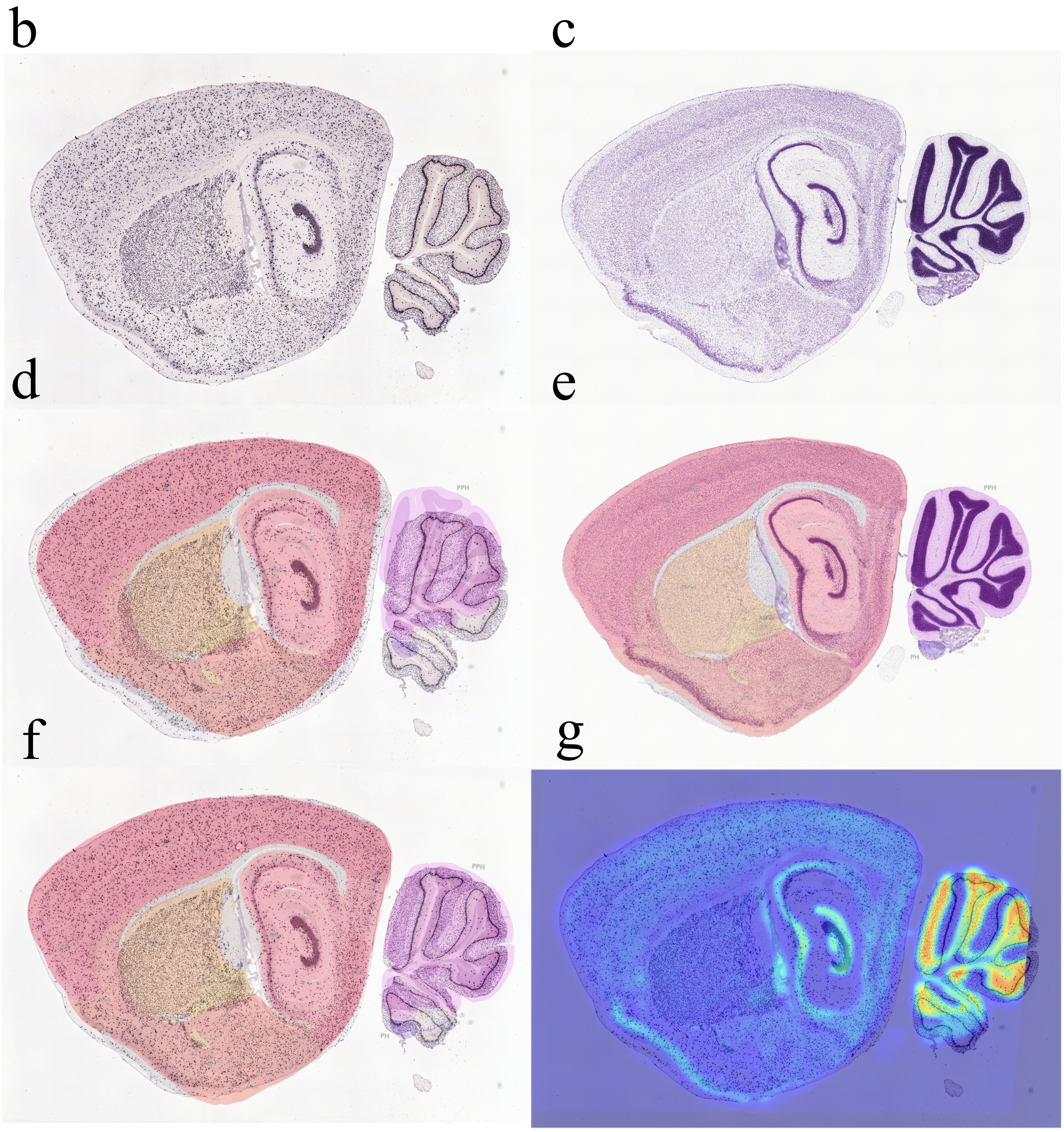

Supplementary Figure 6. Automatic registration of a lateral brain section (sagittal) of an adult brain with Allen brain developing reference atlas. a) Figure shows the first ten iterations by optimization of affine transformation algorithm in ITK toolkit. **b)** Sample lateral (sagittal) section of VGAT marker from P56 mouse brain. **c)** Nissl brain section against the corresponding brain section in (b) from the common coordinate framework in Allen brain atlas. **d)** Overlaid reference atlas on top of the brain section in (b) before registration. **e)** Overlaid reference atlas on top of the pre-registered Nissl brain section. **f)** Overlaid reference atlas on top of the VGAT brain section in (b) after registration. **g)** Overlaid VGAT brain section with transformed Nissl brain section after registration. The original brain sections are obtained from [3]. Image credit: Allen Institute.

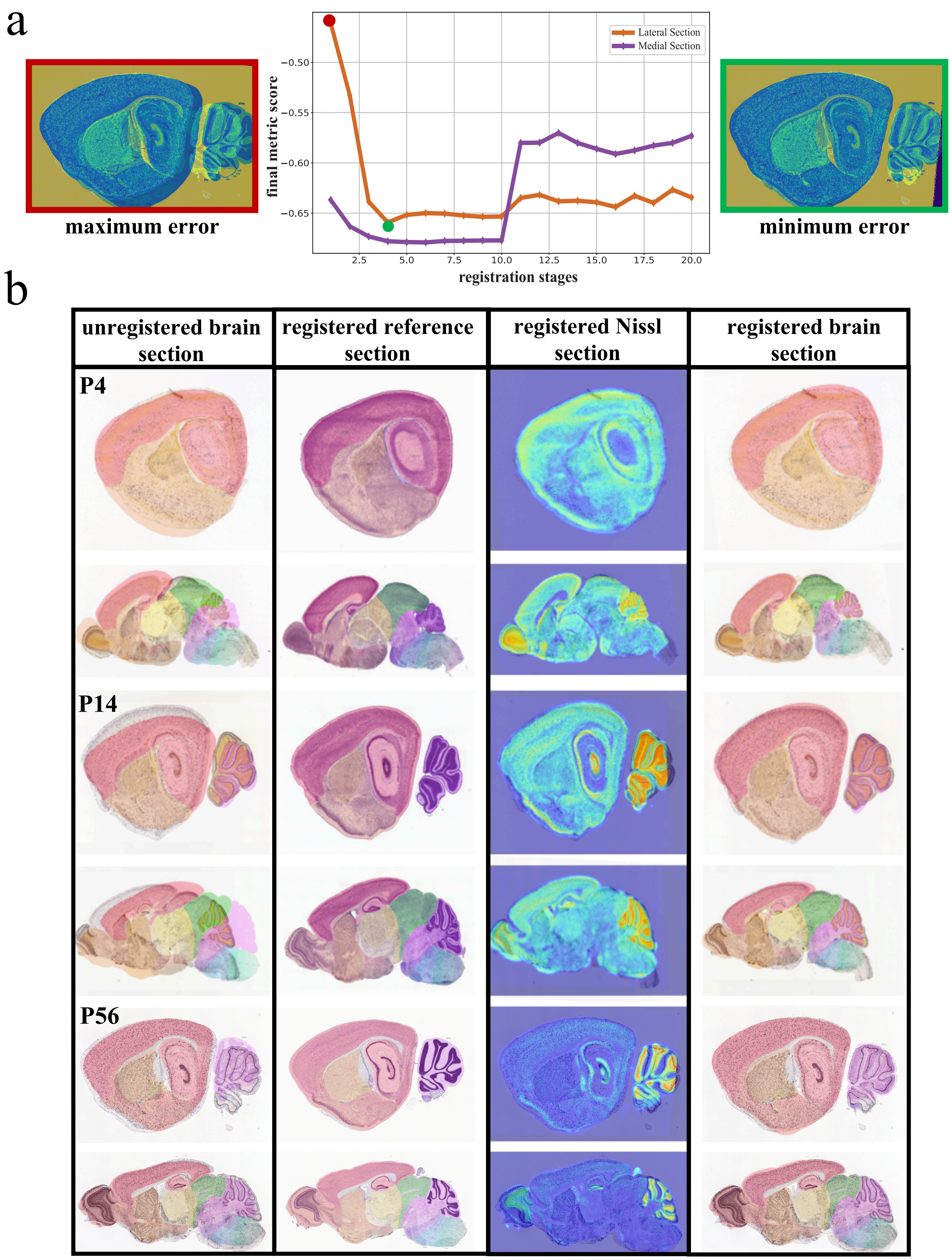

Supplementary Figure 7. Automated Brain Registrator Framework. a) A recurrent strategy is applied to register a given brain section with the corresponding reference atlas through optimization of an affine transformation algorithm. The graph in the middle shows the registration error on two brain sections: 1) lateral (brown) and 2) medial (purple). The registration error usually reaches the minimum in the first ten recurrences. Red and Green points shows the maximum and minimum errors across the same brain section overlaid with its reference atlas on left and right. The registered reference atlas with the minimum error is used for final registration. **b)** Nissl brain sections are pre-registered to the colored reference atlas (column # 2). Performance of brain registration on random samples of P4, P14 and P56 mice brains is shown as before (column#1) and after (column#4) the registration. Nissl section is first transformed to the corresponding brain section (column#3) then afterwards the same transformation is applied to the colored reference atlas (column#4). The original brain sections are obtained from [3]. Image credit: Allen Institute.

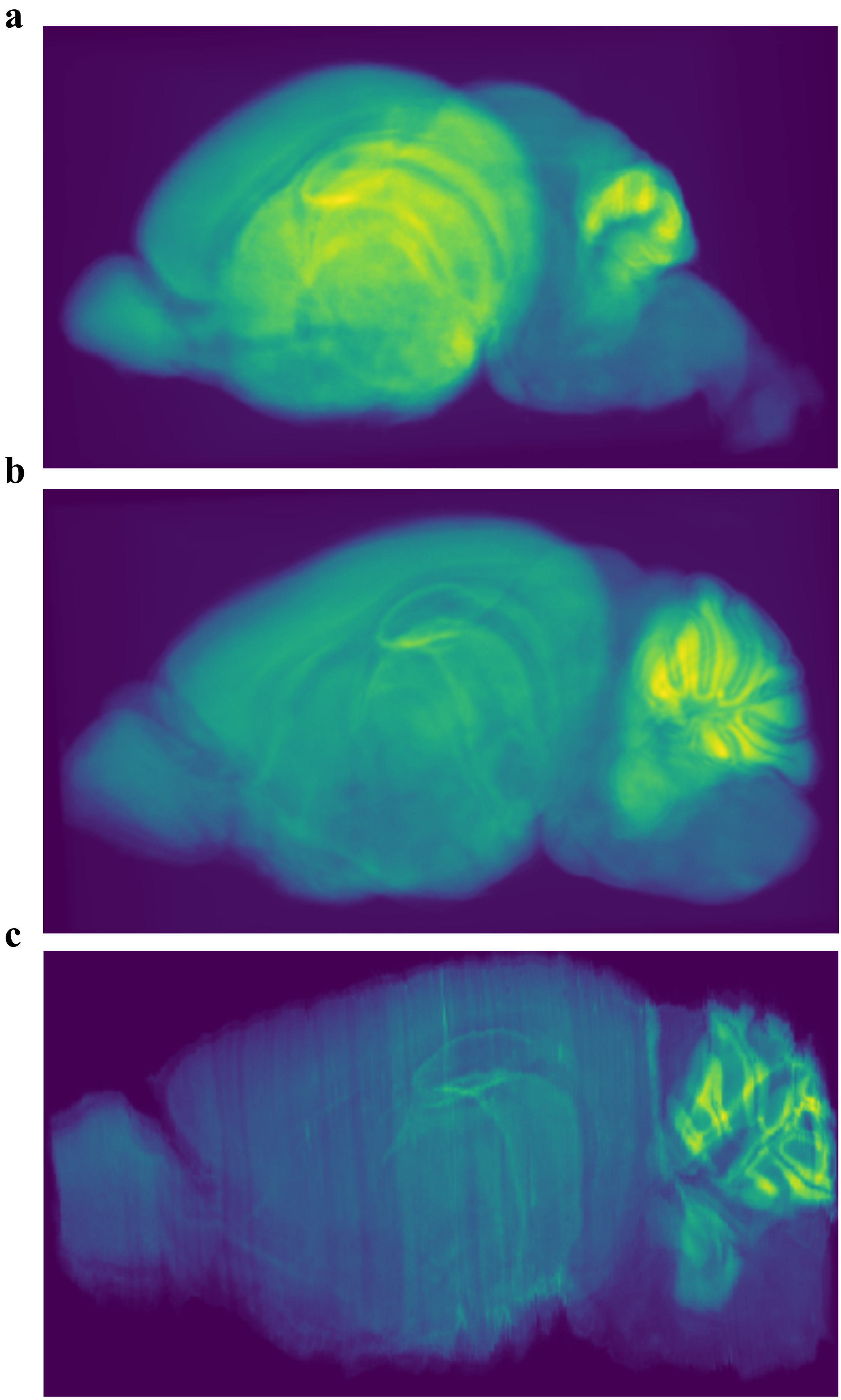

Supplementary Figure 8. 3D Nissl brains of P4, P14 and P56 old mice. a, P4 Nissl mouse brain is shown from the side view and P14 and P56 brains can be visualized in b-c. These Nissl brains are used as reference for automated registration of a given brain section. For every 2D brain section, a corresponding reference atlas in also provided at the Allen Brain Institute.

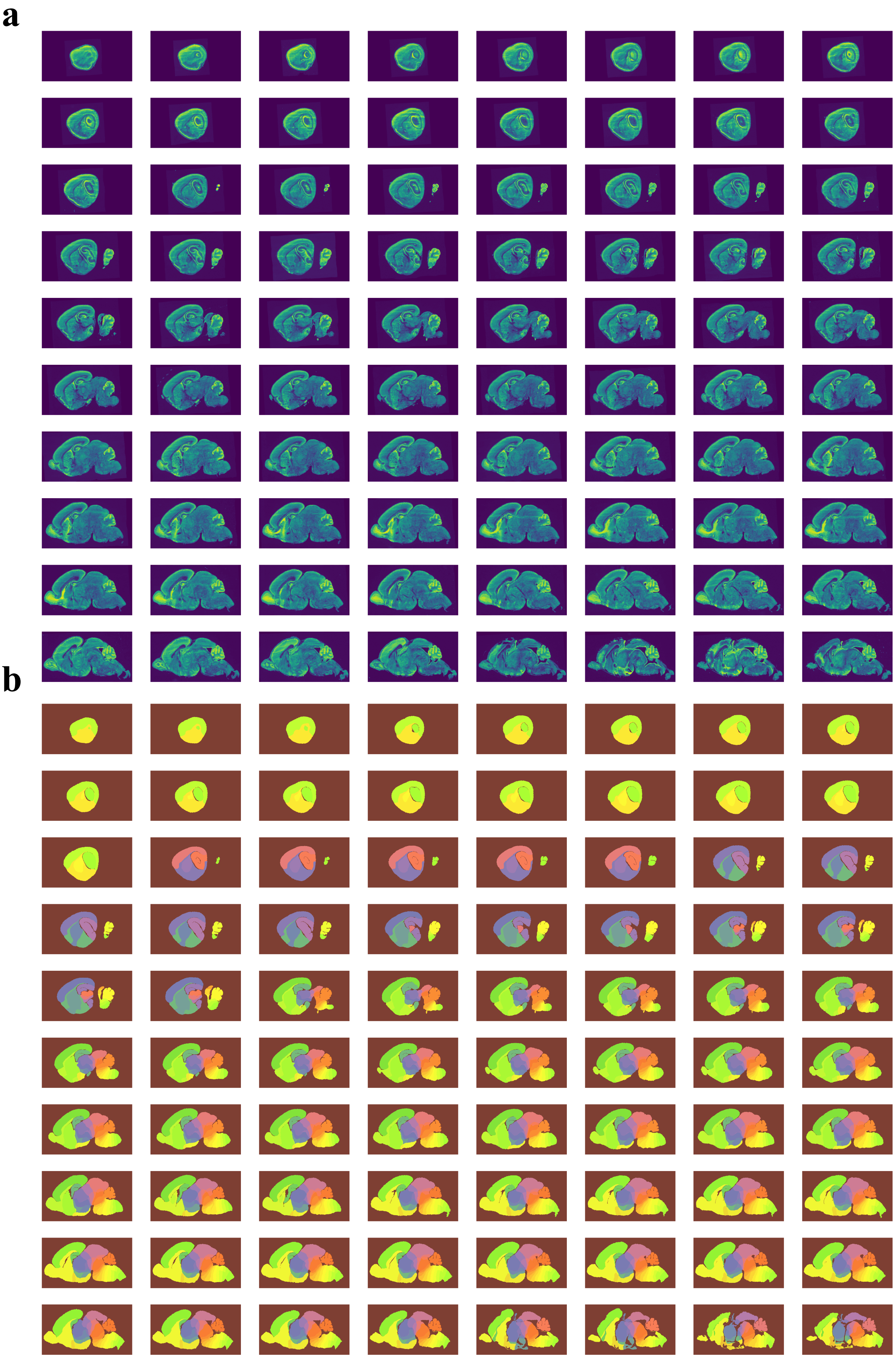

Supplementary Figure 9. Sagittal brain sections of P4 Nissl brain with their corresponding reference atlas. a, Nissl brain sections of P4 mouse brain from sagittal plane (lateral to medial) are shown with their corresponding atlas in (b). These Nissl sections are used for registration of any other brain section for mapping the complete brain regions.

a

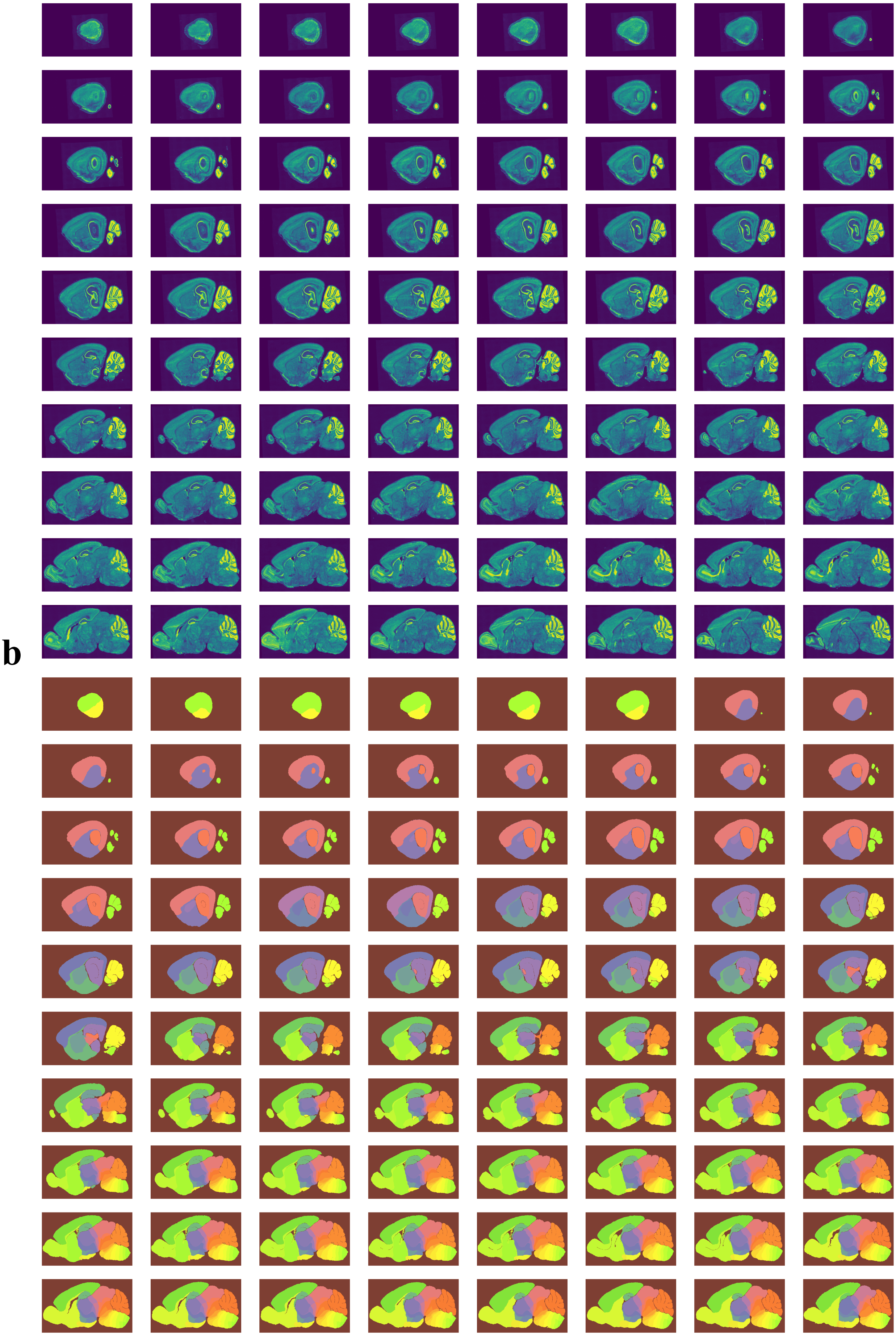

Supplementary Figure 10. Sagittal brain sections of P14 Nissl brain with their corresponding reference atlas. a, Nissl brain sections of P14 mouse brain from sagittal plane (lateral to medial) are shown with their corresponding atlas in (b). These Nissl sections are used for registration of any other brain section for mapping the complete brain regions.

a

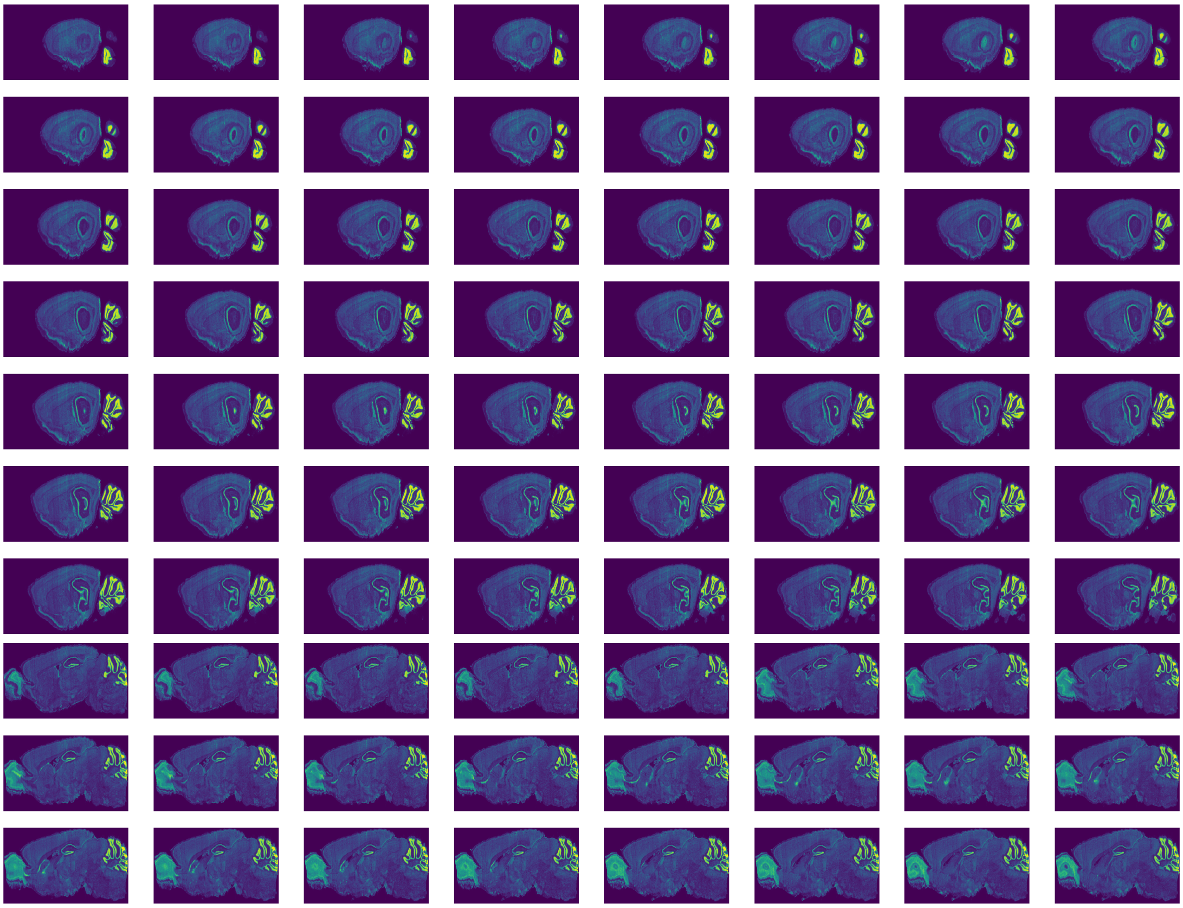

b

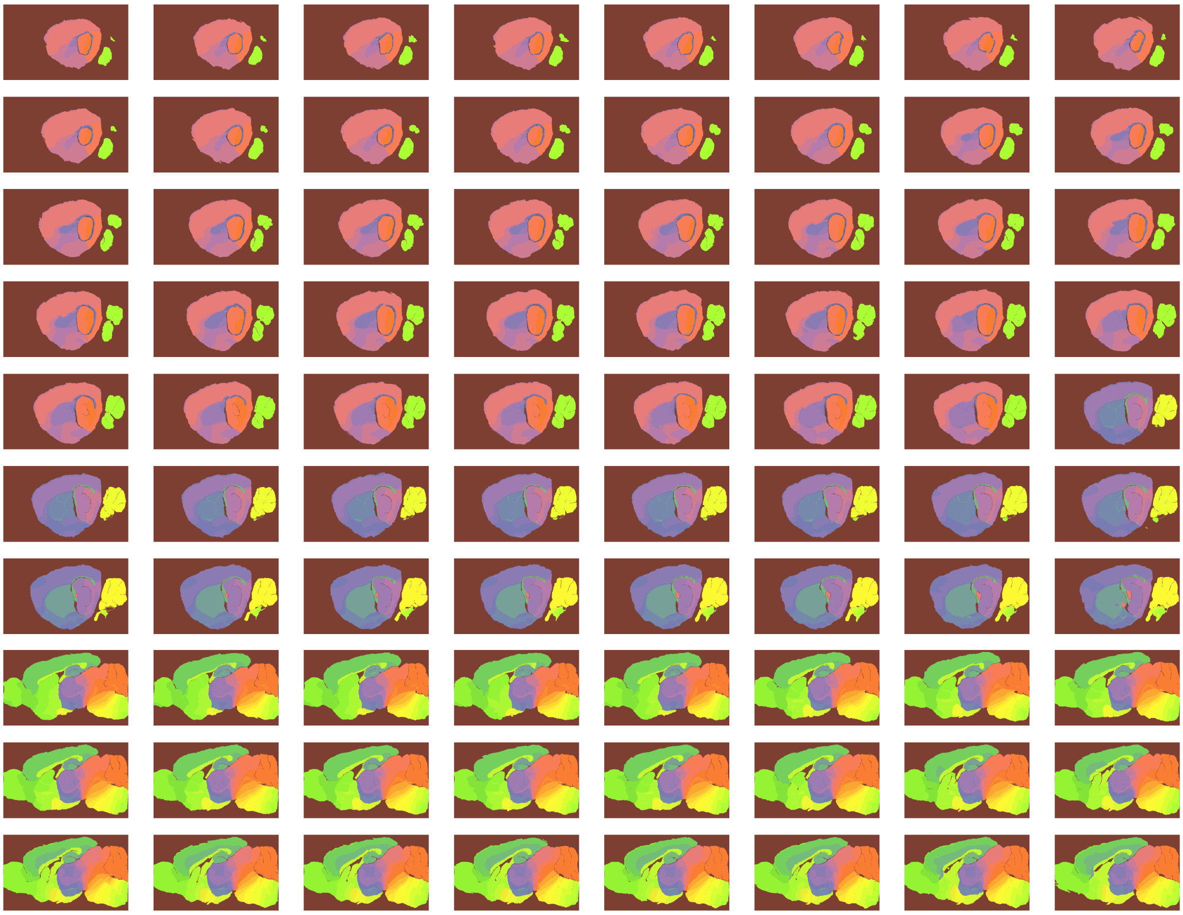

Supplementary Figure 11. Sagittal brain sections of P56 Nissl brain with their corresponding reference atlas. a, Nissl brain sections of P56 mouse brain from sagittal plane (lateral to medial) are shown with their corresponding atlas in (b). These Nissl sections are used for registration of any other brain section for mapping the complete brain regions.
